# Decoupling proton reactivity from Zn^2+^ interfacial electrochemistry through solvent isotope substitution

**DOI:** 10.1073/pnas.2619771123

**Published:** 2026-09-08

**Authors:** Taizhe Liu, Md. Arif Faisal, Ashutosh Rana, Ashutosh Bhadouria, James H. Nguyen, Saptarshi Paul, Brian M. Tackett, Jeffrey E. Dick

**Affiliations:** ^a^ Department of Chemistry, Purdue University, West Lafayette, IN 47907; ^b^ Davidson School of Chemical Engineering, Purdue University, West Lafayette, IN 47907; ^c^ Elmore Family School of Electrical and Computer Engineering, Purdue University, West Lafayette, IN 47907

**Keywords:** aqueous Zn metal batteries, kinetic isotope effect, hydrogen evolution reaction, corrosion, additive

## Abstract

The parasitic hydrogen evolution reaction (HER) and corrosion fundamentally limit the reversibility of aqueous zinc batteries, yet their mechanistic origin remains unresolved. Here, we establish an experimental framework that probes bulk/diffuse-layer electrolyte properties via isotopic substitution (H_2_O → D_2_O) in hydrated zinc electrolytes. This approach largely preserves Zn^2+^-centered interfacial electrochemical behavior, as evidenced by indistinguishable nucleation overpotentials and steady-state fast scan voltammetric responses at ultramicroelectrodes (UMEs), while selectively modulating bulk proton transport properties and HER kinetics. Despite largely preserved interfacial energetics, electrochemical mass spectrometry (ECMS) reveals a pronounced suppression of gaseous side products in D_2_O-based electrolytes, directly implicating bulk and diffuse-layer H/D solvent dynamics as an important control over side reaction kinetics. Notably, this framework reveals that gas evolution emerges at comparable overpotentials across isotopic systems, indicating that the onset of parasitic activity is not governed by independent heterogeneous reaction pathways. Instead, these observations are consistent with a Zn^0^-associated activation process that includes corrosion-mediated HER during zinc electrodeposition, direct cathodic water reduction, or a combination of both pathways. Moreover, these findings reconcile and resolve long-standing observations in the zinc battery field in terms of HER mechanism and the additive literature, where trace components disproportionately enhance stability, by highlighting the critical roles of interfacial adsorption and double-layer structure alongside bulk solvation effects.

## Significance

To meet the global energy demand, new chemistries are required to access grid-level energy storage devices. Aqueous metal batteries, including those made of zinc, are rising as promising candidates owing to their intrinsic safety, high theoretical capacity, and relatively low cost. However, grid-scale deployment is hindered by instabilities at the anode, particularly the mechanistic origin of the hydrogen evolution reaction. Here, through solvent isotopic exchange, we provide evidence that hydrogen evolution is strongly coupled to Zn deposition and is consistent with a substantial spontaneous, corrosion-associated contribution, while involving concurrent cathodic water reduction. These results provide significant and foundational chemical insight to inform on both electrolyte formulations and anode material design.

In recent years, lithium-ion batteries (Li-ion) have dominated the energy storage landscape due to their high energy density and widespread applicability in portable electronics and electric vehicles (1, 2). However, issues such as flammable organic electrolytes, limited lithium resources, and rising costs have spurred interest in alternative chemistries (3). Among emerging candidates, aqueous Zn-metal batteries (AZMBs) offer notable advantages such as high theoretical capacity (820 mAh g^−1^ and 5,854 mAh cm^−3^), low redox potential (−0.76 V vs. SHE), inherent safety, and material abundance, making them a compelling option for cost-effective and sustainable energy storage (4–7). Despite these benefits, their practical implementation is hindered by dendritic Zn growth, parasitic side reactions, and the formation of unstable solid-electrolyte interphases (SEIs) at the anode front (8–10). At the core of these challenges lies the hydrogen evolution reaction (HER), a persistent and detrimental side reaction in mildly acidic aqueous electrolytes (pH 4-6) used for reversible AZMBs (11, 12). HER not only reduces energy efficiency and shortens cycle life but also exacerbates Zn corrosion and passivation (13–15). Although strategies such as electrolyte modification, novel additive formulations, and current collector design have been proposed to mitigate dendrite formation and suppress HER at the anode, the role of solvent proton dynamics in driving these instabilities remains poorly understood (16–20).

HER is an unavoidable parasitic reaction in mildly acidic aqueous systems, and numerous strategies have been developed to suppress it (21–23). In aqueous Zn electrolytes, Zn^2+^ predominantly exists as [Zn(H_2_O)_6_]^2+^, in which water molecules coordinate to the Zn^2+^ center to form the solvation shell. These Zn^2+^-bound water molecules are acidified by the strong Lewis-acid polarization of Zn^2+^, making them more susceptible to interfacial proton-coupled reduction than bulk/free water (21, 24–26). Accordingly, much of the community has focused on reducing the amount of water in the Zn^2+^ solvation sheath and increasing the desolvation barrier to suppress HER (27–30). However, although Zn^2+^-coordinated water is more chemically activated, free water proton dynamics and interfacial H-bonded water still govern how effectively that activation is translated into HER (7, 31, 32). Thus, HER associated with free water cannot be neglected. Within this framework, two distinct but related parasitic processes should be considered. The first is resting loss (calendar aging), in which deposited Zn is spontaneously consumed during idle periods through galvanic corrosion coupled to HER. Researchers have reported that resting loss increases with electrolyte acidity and with the electrocatalytic affinity of the current collector. Thermodynamically, this process is expected because the Zn^2+^/Zn redox couple (−0.76 V vs. SHE) lies far below the H^+^/H_2_ couple (0 V vs. SHE), rendering proton reduction more favorable than Zn metal stability in water. Consequently, deposited Zn is never truly at rest: Zn^0^ can spontaneously oxidize, while the released electrons are consumed by hydrogen evolution on any electrochemically connected conductive surface (33–37). The second is faradaic HER from bulk water, in which electrons supplied for Zn deposition are partially diverted to water reduction under cathodic bias. Unlike resting loss, this process is externally driven by the applied current (7, 32, 38, 39). However, in mildly acidic or near-neutral Zn electrolytes, both pathways can generate H_2_ and OH^−^, and the resulting local alkalization promotes the formation of passivating byproducts such as Zn hydroxides and zinc hydroxide sulfate (9, 10, 35, 38). Therefore, they not only lower coulombic efficiency (CE) but also aggravate interfacial instability and nonuniform Zn growth by continuously perturbing the local chemical environment and the homogeneity of the electric field (40). Although these two processes occur under different conditions, both are ultimately sustained by proton transfer and solvent reorganization in water (25, 32, 39).

To suppress HER, the field has largely relied on electrolyte-engineering strategies that regulate water activity and perturb the hydrogen-bond network. However, these effects are often inseparable from changes in Zn^2+^ solvation energetics or electrical-double-layer structure (21, 41, 42). For example, pentaerythritol was introduced to disrupt the hydrogen-bond network of water, yet the same study also showed a changed Zn^2+^ solvation structure (43). Similarly, weakly solvating cosolvent strategies suppress water-mediated side reactions partly by reducing interfacial water without directly targeting solvation energetics, but they also increase anion participation in the Zn^2+^ solvation sheath, thereby altering desolvation behavior (44, 45). Even ectoine, which was designed to impede proton hopping by strengthening hydrogen-bond interactions, did not act exclusively on bulk water: a small fraction of ectoine entered the Zn^2+^ solvation environment and partially displaced SO_4_^2−^ coordination (46). As a result, although many studies report suppressed HER, it remains unclear whether the dominant benefit arises from modifying the Zn^2+^ interfacial environment or from slowing the bulk water dynamics that sustain proton transport and water reduction. This aspect represents a critical knowledge gap in the literature and motivates the development of an experimental platform to systematically probe HER suppression governed by solvation energetics and the structure of the bulk water network (47–49).

In this work, we show that D_2_O substitution in the ZnSO_4_‧7H_2_O system provides an effective platform to probe the role of bulk and diffuse-layer water dynamics in HER. This simple yet powerful solvent-isotope substitution markedly reduces hydrogen evolution, promotes more uniform Zn deposition, and greatly improves cycling stability. Although performance enhancement is not the primary focus of this work and the beneficial effects of D_2_O have been previously reported, the mechanism by which D_2_O enhances Zn-metal battery performance remains largely speculative, and more importantly, the role of the bulk solvent network in mediating this behavior is not well understood (50). This limitation motivated the development of an experimental platform based on isotopic substitution of the aqueous electrolyte matrix to distinguish contributions from Zn^2+^ solvation energetics and the surrounding water network. The results presented herein also reveal an overlooked aspect of the zinc battery additive literature. While trace amounts of additives have been reported to dramatically enhance stability through solvation modulation, such effects are difficult to rationalize on the basis of bulk mole fraction alone. Our findings instead suggest that additive adsorption and modulation of the electrical double layer (EDL) may play an important role in governing zinc anode stability. Here, we combined spectroscopic, electrochemical, and structural characterizations to probe the role of free water in HER and clarify how AZMB CE can be improved through regulation of solvent reorganization and bulk/diffuse-layer proton transport properties. We first employed ^67^Zn NMR and fast-scan cyclic voltammetry (FSCV) on tungsten ultramicroelectrodes (UMEs) to confirm that Zn^2+^ solvation energetics remain largely unchanged after D_2_O substitution. Combined with ^17^O NMR, FTIR, and C_dl_ measurements, these results lead us to propose that crystal water derived H_2_O from salt helps preserve a similar Zn^2+^ interfacial environment, whereas the D_2_O-rich bulk phase suppresses hydrogen bond-mediated water dynamics. We then used electrochemical mass spectrometry (ECMS) to show that total gas evolution is markedly reduced in D_2_O-containing systems, both in the presence and absence of Zn salt, underscoring the important role of solvent-network suppression in water-mediated reactivity. Isotope-resolved ECMS further reveals that in Zn-free acidic electrolytes, H_2_, HD, and D_2_ display staggered onset behavior, whereas in ZnSO_4_ electrolytes all isotopic gas products emerge together with Zn deposition. This common onset of all three isotopologues provides a stringent validation of Zn-gated HER activation: Parasitic gas evolution is not simply a set of independent acid-like HER/HDER/DER pathways but is coupled to Zn deposition and Zn-mediated interfacial/corrosion chemistry. At the same time, the D-rich solvent network lowers the H/D donor flux that sustains gas evolution, thereby suppressing both faradaic and corrosion-mediated side reactions. Finally, Zn symmetric and asymmetric coin cells with D_2_O-based electrolytes exhibit dramatically prolonged cycling lifetimes exceeding 3,600 and 4,500 h, respectively, at 1 mA cm^−2^ and 0.5 mAh cm^−2^. In addition, even with a 12 h rest between cycles, the initial and average Coulombic efficiencies are increased by 30% and 10%, respectively. X-ray diffraction (XRD) and scanning electron microscopy (SEM) further reveal suppressed passivation byproduct formation and denser, more compact Zn deposits in D_2_O-based electrolytes. Altogether, these results show that D_2_O improves Zn reversibility by primarily attenuating bulk/diffuse-layer water. By slowing proton-coupled solvent reorganization, D_2_O mitigates both resting-loss related side reactions and faradaic HER, thereby reducing byproduct formation and enabling smoother, more stable Zn deposition. Moreover, these findings support a unifying framework in which galvanic corrosion processes make an important contribution to parasitic reactions during zinc battery cycling (51, 52). Broadly, this work identifies bulk-water dynamics as an important yet often overlooked design parameter for stabilizing Zn metal anodes in aqueous electrolytes.

## 1. Results and Discussion

To elucidate how solvent isotope substitution regulates parasitic gas evolution and Zn anode stability, the results are organized into six interconnected sections. We begin by outlining a working framework in which D_2_O substitution strongly perturbs the H/D-containing solvent network while the Zn^2+^-centered electrochemical environment remains largely preserved within experimental resolution. We then examine the Zn-centered coordination/interfacial environment and the surrounding solvent network using complementary spectroscopic and electrochemical measurements to determine whether isotope substitution primarily alters Zn^2+^ electroreduction behavior or H/D solvent dynamics. Next, we employ simplified acidic electrolytes to probe the intrinsic isotope effect on proton/deuteron transport and hydrogen evolution in the absence of Zn. In this Zn-free system, the isotopic gas-evolution channels exhibit staggered onset behavior, consistent with the different kinetic accessibilities of H_2_, HD, and D_2_ formation. We then return to the ZnSO_4_ electrolyte, where Zn deposition and parasitic gas evolution occur at the same electrode, to determine whether isotope-dependent suppression of H/D evolution remains operative under practical Zn plating conditions. In contrast to the acid system, all isotopic gas products in the D_2_O-based ZnSO_4_ electrolyte emerge at the same onset potential. This behavior indicates that water coreduction in the Zn salt electrolyte is not activated as a set of independent acid-like HER/HDER/DER pathways, but is instead coupled to the Zn deposition, appearance of Zn^0^, and the resulting Zn-mediated interfacial/corrosion chemistry. This observation strengthens our previous finding that Zn deposition and hydrogen evolution share the same onset potential: In the present isotope-resolved platform, not only H_2_ but all H/D gaseous products emerge together with Zn deposition, making a coincidental overlap of separate reactions unlikely (26). To the best of our knowledge, the hydrated ZnSO_4_/D_2_O platform provides a uniquely stringent way to validate this prior same-onset finding because it allows multiple isotopically distinct gas products (H_2_, HD, and D_2_) to be monitored simultaneously. We then correlate these mechanistic insights with Zn cycling stability, resting-loss-sensitive CE, structural evolution, and deposition morphology. Finally, we conclude with an integrated discussion that reconciles the present solvent-isotope framework with our prior mechanistic studies on Zn^2+^-associated water reduction, current partitioning, and interfacial reaction-transport feedback (26, 53). Together, these results present HER in aqueous Zn batteries as a coupled, regime-dependent process in which Zn deposition gates the onset of parasitic gas evolution, Zn^2+^ electroreduction kinetics and interfacial feedback regulate current partitioning during plating, while H/D-containing donor transport through the bulk/diffuse layer controls the sustained HER/HDER/DER flux.

### 1.1. Schematic Framework for Solvent-Isotope-Regulated HER Suppression.

In AZMB systems, both galvanic and faradaic HER are widely recognized as key contributors to parasitic side reactions. Galvanic HER occurs when deposited Zn is spontaneously oxidized and the released electrons are consumed by H-containing species on electronically connected surfaces. Faradaic HER occurs when externally supplied electrons are diverted from Zn^2+^ reduction to water reduction during cathodic polarization. Although these two processes occur under different electrochemical conditions, both require interfacial H^+^ transfer, solvent reorganization, and continuous replenishment of reactive hydrogen donors from the surrounding electrolyte (38).

Compared with H_2_O, D_2_O exhibits slower Grotthuss-type transport, slower hydrogen-bond rearrangement, and larger solvent-reorganization barriers, which can impose kinetic penalties on proton/deuteron transfer and water reduction. Therefore, isotopic substitution provides a route to suppress both resting-loss-related galvanic HER and faradaic HER (50, 54, 55). In the ZnSO_4_‧7H_2_O system, crystal-water-derived H_2_O introduces H-containing isotopologues into the nominal D_2_O electrolyte after H/D exchange, primarily as HOD together with residual H_2_O. Because H_2_O exhibits faster dielectric relaxation, a more rapid orientational response, and higher polarizability, it can be preferentially accommodate in regions requiring rapid solvent reorganization, such as the EDL, this creates a dynamically exchanging H_2_O/HOD/D_2_O solvent network. Within this network, under applied bias, highly reactive H-containing species remain available near the Zn^2+^-centered interfacial region, while the less reactive D-containing species are accumulated and retained in surrounding bulk and exhibits slower H/D transport and solvent reorganization (56, 57). Based on this framework, isotope substitution perturbs the H/D-containing solvent network while the Zn^2+^-centered electrochemical environment remains largely preserved within experimental resolution.

In H_2_O-based ZnSO_4_ electrolytes, rapid hydrogen-bond rearrangement and proton hopping facilitate efficient transport of hydrogen donors to the interface, contributing to both spontaneous corrosion and current-driven hydrogen evolution. Moreover, under cathodic bias, bulk free water can participate in interfacial reduction, where its fast reorganization kinetics enable substantial HER via conventional Volmer pathways, leading to byproduct formation and reduced stability (58).

**Fig. 1.**
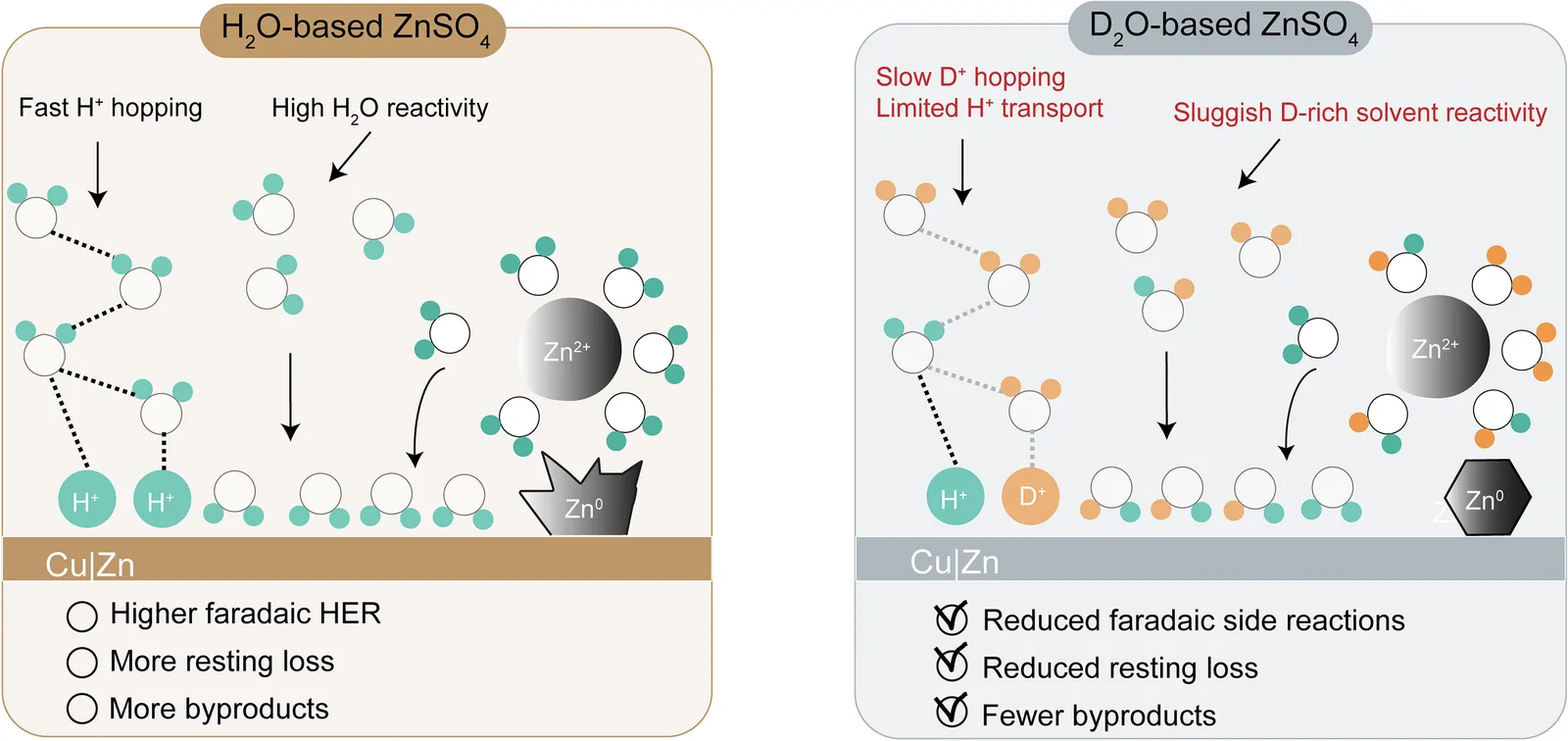
Schematic illustration of the proposed mechanism in H_2_O- and D_2_O-based ZnSO_4_ electrolytes. Green spheres represent H^+^, while orange spheres represent D^+^.

By contrast, in D_2_O-based electrolytes, slower deuteron transport and hindered hydrogen-bond reorganization impose kinetic limitations on H^+^/D^+^ transfer and water reduction, particularly for D-containing solvent species supplied from the bulk electrolyte. At the same time, crystal-water-derived H-containing isotopologues help buffer the Zn^2+^ coordination and near-interfacial environment, allowing Zn^2+^ electroreduction to remain largely preserved. As a result, under cathodic bias, the H-containing isotopologues are primarily delivered to the interface and reduced; however, their continued consumption is coupled to replenishment, H/D exchange, and solvent reorganization within the surrounding D-rich bulk. Because the D-rich solvent network slows H/D transport and solvent reorganization, the overall flux of parasitic gas evolution is suppressed. Thus, isotope substitution suppresses parasitic gas evolution without substantially slowing Zn deposition kinetics. This schematic framework is presented in Fig. 1 and examined experimentally in the following sections through Zn^2+^ electroreduction measurements, bulk H/D transport analysis, isotope-resolved gas evolution, and Zn cycling behavior.

### 1.2. Largely Preserved Zn^2+^ Interfacial Electrochemistry under Bulk-Solvent Perturbation.

**Fig. 2.**
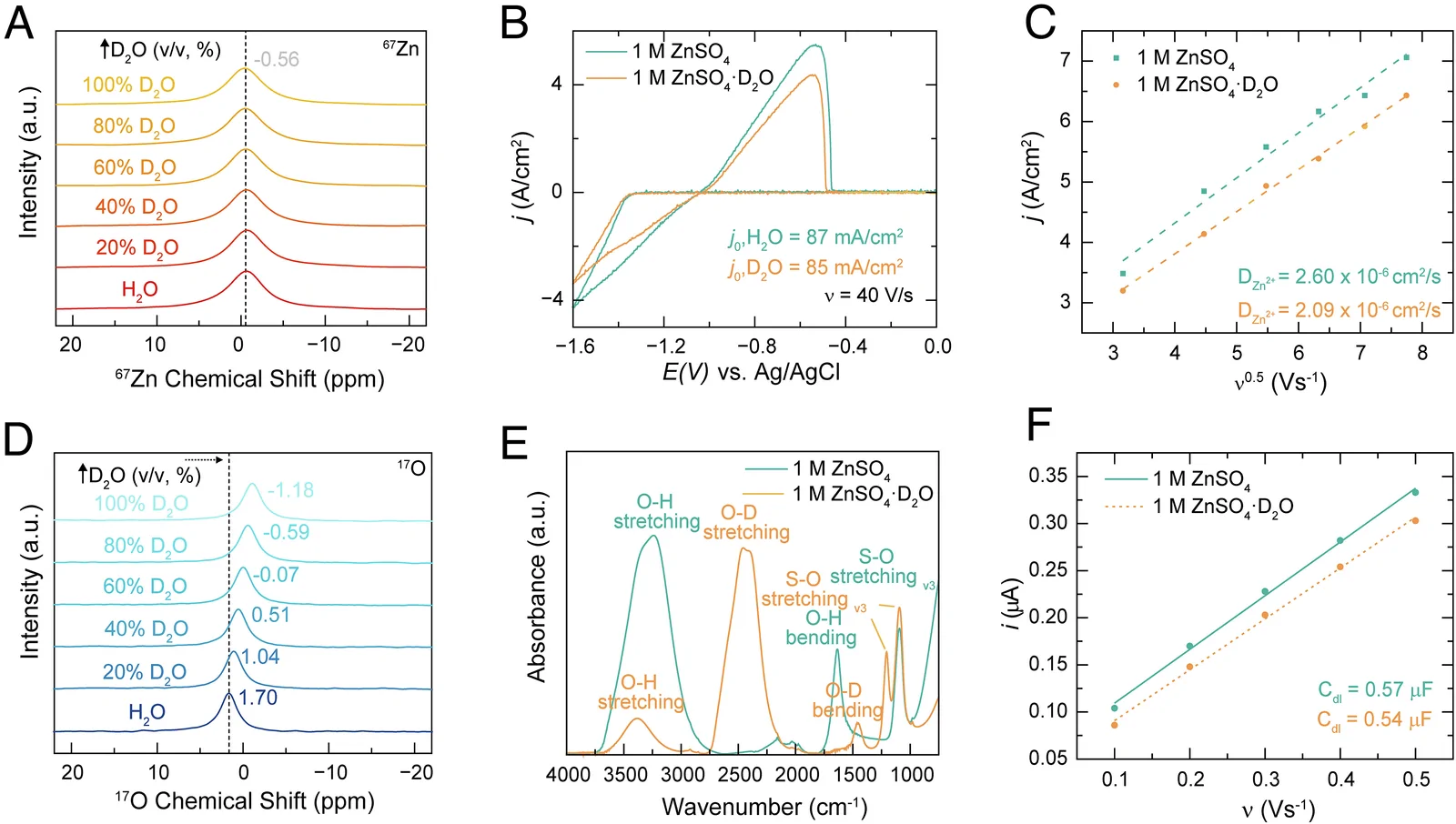
(*A*) ^67^Zn NMR spectra of 1 M ZnSO_4_ electrolytes prepared with increasing D_2_O fraction (0 to 100 vol%). (*B*) Overlay of cyclic voltammograms of 1 M ZnSO_4_ in H_2_O and D_2_O on a two-electrode setup with a 12.5 μm radius tungsten microelectrode (UME) as working electrode and Ag/AgCl in 1 M KCl as the reference electrode at a scan rate of 40 V s^−1^. Tafel analysis within kinetic-controlled window yields *j_0_* of 87 and 85 mA cm^−^^2^ for H_2_O- and D_2_O-based electrolyte. (*C*) Diffusion coefficient ($D_{{\mathrm{Zn}}^{2+}}$) of Zn^2+^ in H_2_O and D_2_O in 1 M ZnSO_4_, derived from FSCV on tungsten UME. (*D*) ^17^O NMR spectra of 1 M ZnSO_4_ electrolytes prepared with increasing D_2_O fraction (0 to 100 vol%). (*E*) FTIR spectra for 1 M ZnSO_4_ in H_2_O and D_2_O. (*F*) Double-layer capacitance (C_dl_) extracted from cyclic voltammetry on glassy carbon electrodes.

To experimentally probe both the Zn^2+^-coordinated environment and the surrounding solvent network, a series of 1 M ZnSO_4_‧7H_2_O electrolytes containing 0%, 20%, 40%, 60%, 80%, and 100% D_2_O/H_2_O were prepared. As shown in Fig. 2*A*, ^67^Zn NMR exhibits essentially no measurable shift with increasing D_2_O content, indicating that the immediate Zn^2+^ coordination environment remains largely unchanged. To further evaluate whether isotopic substitution affects Zn^2+^ solvation energetics, we next examined the electrochemical kinetics of Zn deposition. FSCV was performed using a lab-made tungsten ultramicroelectrode with a radius of 12.5 μm. Compared with conventional macroelectrodes, UMEs promote semi-infinite radial diffusion and substantially reduce capacitive currents and iR drop, thereby enabling kinetically controlled electrodeposition behavior to be probed more directly with less interference from mass transport and side reactions (59, 60). Across a series of scan rates, the extracted exchange current density within kinetically controlled regime became effectively scan-rate independent beginning at 40 V s^−1^ and remained similar from 40 to 60 V s^−1^, indicating that contributions from mass transport and side reaction were minimized in this regime (*SI Appendix*, Table S1). We therefore selected 40 V s^−1^ as the representative fast-scan condition for kinetic comparison. As shown in Fig. 2*B*, the H_2_O- and D_2_O-based ZnSO_4_ electrolytes display highly similar current-potential profiles with well-defined Zn plating and stripping features. Tafel analysis of the backward scan (*SI Appendix*, Fig. S1) yields nearly identical exchange current densities (*j*_0_) of 87 mA cm^−2^ for H_2_O and 85 mA cm^−2^ for D_2_O, confirming that the charge-transfer kinetics of Zn^2+^ reduction remain essentially unchanged after isotopic substitution. However, despite comparable charge-transfer kinetics (also evidenced by overlapping currents near the equilibrium potential in Fig. 2*B*), the reduced peak current observed in both cathodic and anodic regimes likely arise from differences in bulk transport properties (proton/hydronium transport), as discussed below. We further examined Zn^2+^ transport through diffusion coefficients extracted from FSCV. As shown in Fig. 2*C* and *SI Appendix*, Fig. S2, only a minor difference in $D_{{\text{Zn}}^{2+}}$ is observed between the H_2_O and D_2_O systems, indicating that isotopic substitution does not significantly perturb Zn^2+^ mass transport under these conditions (61, 62). Taken together, the unchanged ^67^Zn NMR response, nearly identical exchange current densities, and similar Zn^2+^ diffusion behavior indicates that the fundamental dynamics of Zn^2+^ deposition remain largely preserved between the two electrolytes within experimental resolution.

Having established that the Zn^2+^-coordinated electrochemical environment is largely preserved, we next turned our attention to the surrounding solvent network. In contrast to the unchanged ^67^Zn response, the oxygen environment shows a clear isotopic dependence. As displayed in Fig. 2*D*, ^17^O NMR reveals a progressively more shielded oxygen signal with increasing D_2_O content, corresponding to the weakened hydrogen-bonding in the bulk electrolyte. This distinction between Zn-centered invariance and oxygen-centered variation suggests that isotopic substitution more strongly affects the surrounding bulk solvent network than the primary Zn^2+^ coordination sphere. Thus, while the immediate Zn^2+^ environment remains effectively unchanged, the collective solvent dynamics are clearly modified. Notably, FTIR in Fig. 2*E* confirms the coexistence of D-containing and residual H-containing species in the D_2_O-based electrolyte. In particular, the split peaks at 1,089 and 1,209 cm^−1^ assigned to the asymmetric stretching mode of SO_4_^2−^, together with the O–H stretching signatures in the D_2_O-based electrolyte, indicate that H-containing isotopologues remain present. These H-containing species most plausibly originates from two unavoidable sources: the hygroscopic uptake of atmospheric H_2_O during D_2_O handling and the crystal water introduced by the ZnSO_4_‧7H_2_O precursor (50). However, best efforts were made to minimize handling-related errors.

These observations motivated us to further probe the interfacial environment through double-layer capacitance (C_dl_), open-circuit potential (OCP), and crossover potentials extracted from slow-scan cyclic voltammetry (SSCV) and FSCV. C_dl_ was determined from the slope of i_pc_ vs. scan rate using a glassy carbon macroelectrode over a wide scan-rate range. As shown in Fig. 2*F*, the two electrolytes exhibit very similar C_dl_ values of 0.57 and 0.54 μF. Likewise, the Zn OCP and crossover potentials derived from SSCV and FSCV (*SI Appendix*, Table S2) differ by no more than 4 mV between 1 M ZnSO_4_ in H_2_O and D_2_O. Although increasing scan rate shifts the crossover cathodically by approximately 20 to 25 mV because of suppressed corrosion at fast scan rates, the extent of this shift is essentially identical in both solvents. These results indicate that isotopic substitution has minimal influence on the Zn^2+^/Zn equilibrium potential. Collectively, the evidence from Zn NMR, *j_0_*, C_dl_, OCP, and crossover analysis supports the same conclusion: the Zn^2+^ solvation and interfacial environment remains largely unchanged after replacing H_2_O with D_2_O in 1 M ZnSO_4_ electrolyte. In contrast, the ^17^O NMR and FTIR indicate that the bulk solvent environment is clearly altered. Combined with literature showing that H_2_O has faster dielectric relaxation, more rapid orientational response and higher polarizability than D_2_O, these results support our working hypothesis that H-containing isotopologues retain a kinetic advantage in approaching the Zn^2+^ interfacial region, whereas D_2_O exerts a stronger influence on the bulk solvent transport and reorganization. In this sense, the ZnSO_4_‧7H_2_O system provides a useful platform for distinguishing Zn^2+^-centered electroreduction behavior from H/D solvent-network dynamics.

At this point, a simple mass-balance estimate clarifies the hydrogen inventory in the nominal D_2_O electrolyte. Because the electrolyte is prepared from 1 M ZnSO_4_‧7H_2_O, the salt contributes 7 mol L^−1^ of H_2_O equivalents. After mixing with D_2_O, these waters are expected to undergo isotopic scrambling through $H_{2}O+D_{2}O⇌2HOD$, and so the bulk phase is better described as a D_2_O/HOD/H_2_O mixture. Using an effective D_2_O concentration of approximately 50 M and $K_{eq}\approx 3.8$, the equilibrated composition is estimated to contain only ~1 to 2% H_2_O but a much larger HOD fraction of ~20 to 22%. Thus, the D_2_O-based ZnSO_4_ electrolyte contains a substantial reservoir of H-containing isotopologues embedded within a D-rich bulk solvent network. While these isotopologue fractions are theoretical equilibrium estimates based on the total H_2_O/D_2_O inventory and the assumption of complete isotopic scrambling, this theoretically estimated dynamic H/D solvent composition is consistent with the continued availability of H-containing donors near the interfacial region, while the surrounding D_2_O-rich bulk retains slower transport and reorganization dynamics. During electrochemical polarization, preferential consumption of H-containing species can locally perturb the interfacial isotopologue distribution. However, nearby H_2_O/HOD species can continuously re-equilibrate, preventing H depletion from proceeding as a simple one-way process. Moreover, under the Zn deposition conditions examined here, the extent of proton consumption is small relative to the total hydrate-derived proton inventory. In our previous ECMS measurements at 1.5 mA cm^−2^ for 600 s, only 0.3% of the total charge was attributed to HER. Thus, electrochemical consumption of H-containing water is slow relative to the available H reservoir, allowing the dynamic interfacial H/D balance to be sustained over the timescale of Zn deposition. This interpretation is further supported by the isotope-resolved ECMS measurements discussed in the following section (26, 63, 64).

To further test the role of structural water, we compared the FSCV-derived *j_0_* (*SI Appendix*, Table S3) using nonhydrated Zn(OTf)_2_, which lacks the crystal water present in ZnSO_4_‧7H_2_O. In that case, a modest but clear shift in *j_0_* is observed as the solvent is changed from 100% D_2_O to a 50/50 H_2_O/D_2_O mixture, whereas only a negligible additional shift occurs upon further changing the solvent from 50/50 H_2_O/D_2_O to 100% H_2_O. This contrast suggests that when structural water is absent, D_2_O can more readily perturb Zn^2+^ electroreduction energetics. Although replacing ZnSO_4_ with Zn(OTf)_2_ also changes the anion identity and possible ion-pairing behavior, both salts under water-rich conditions are expected to maintain a predominantly hydrated Zn^2+^ first solvation shell. Thus, this comparison supports the broader interpretation that crystal-water-derived H-containing isotopologues in ZnSO_4_‧7H_2_O help buffer the Zn^2+^-centered electrochemical environment. By comparison, the largely preserved *j*_0_ in the ZnSO_4_‧7H_2_O system indicates that the dominant isotope effect resides in the H/D solvent network rather than in a substantial slowing of Zn^2+^ charge transfer. This solvent-network effect is examined more directly in the following section.

### 1.3. Sluggish Deuteron Dynamics Suppress H/D Evolution in the Absence of Zn.

Having shown that the Zn^2+^-centered electrochemical environment is largely preserved while the surrounding solvent network is strongly perturbed in the ZnSO_4_‧7H_2_O system, we next sought more direct mechanistic evidence for the isotope-dependent suppression of H/D transfer. The preceding results indicate altered hydrogen-bond-mediated solvent dynamics, but they do not by themselves reveal how D_2_O affects proton/deuteron reduction in the absence of Zn^2+^ solvation and Zn electrodeposition. This distinction is important because both faradaic HER during Zn deposition and spontaneous corrosion-mediated HER during rest ultimately require H/D transfer and solvent reorganization. We therefore turned to a simplified acidic electrolyte, where proton/deuteron reduction can be examined without the added complexity of Zn^2+^ solvation, Zn nucleation, and Zn-mediated corrosion. By combining proton/deuteron diffusion measurements with ECMS quantification of isotopic gas evolution, we directly probe the kinetic penalty imposed by deuterium substitution on H/D gas evolution in the absence of Zn.

**Fig. 3.**
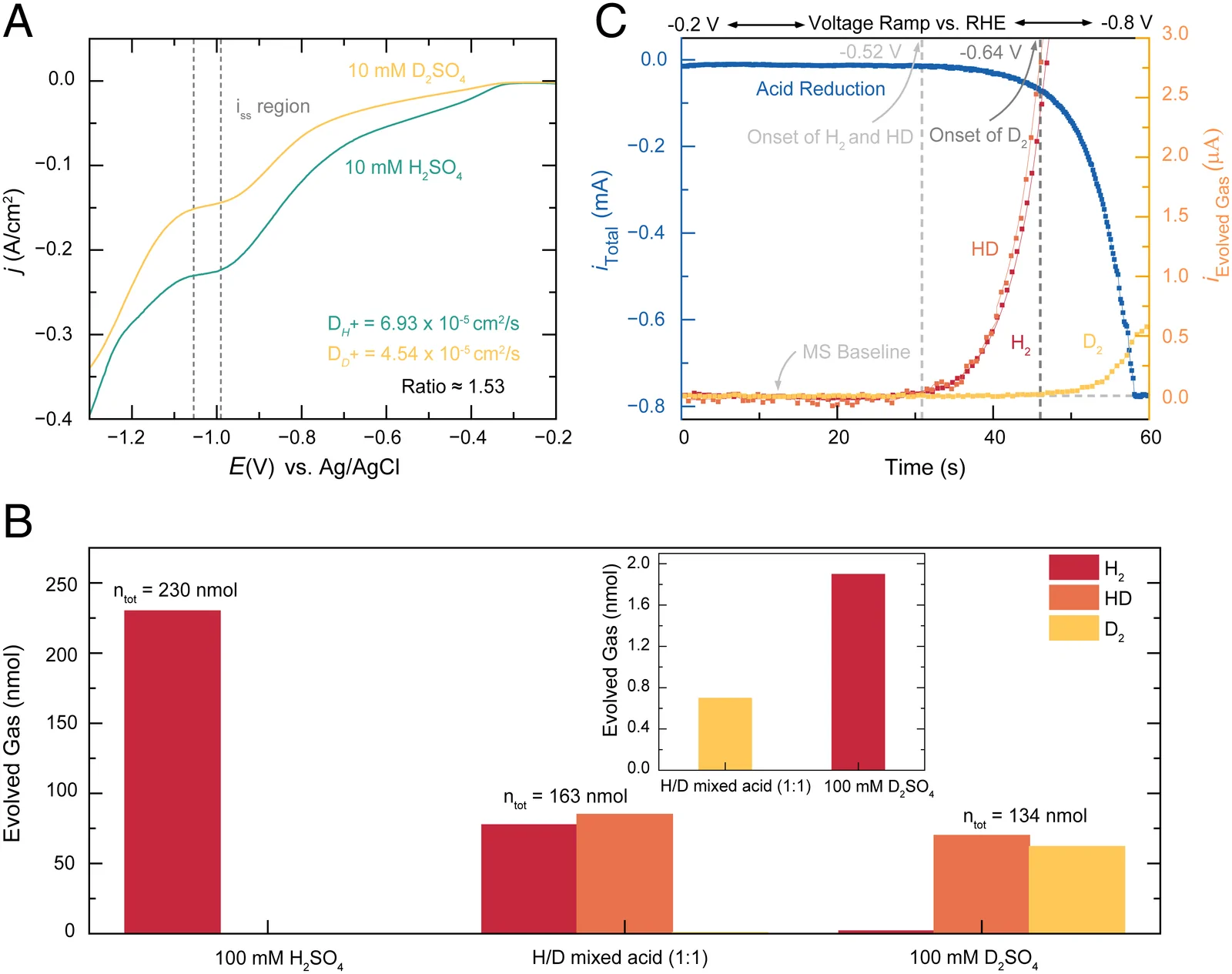
(*A*) LSV (ν = 5 mV s^−1^) recorded on a Pt ultramicroelectrode in 10 mM H_2_SO_4_ and 10 mM D_2_SO_4_ (vs. Ag/AgCl). The steady-state diffusion-limited currents yield proton and deuteron diffusion coefficients of 6.93 × 10^−5^ and 4.54 × 10^−5^ cm^2^ s^−1^, respectively, corresponding to a $D_{H^{+}}/D_{D^{+}}$ ratio of 1.53. (*B*) Quantification of evolved gas measured by ECMS during galvanostatic polarization at -0.2 mA on a Cu working electrode in 100 mM H_2_SO_4_, a 1:1 mixed H/D acid (50 mM H_2_SO_4_+ 50 mM D_2_SO_4_), and 100 mM D_2_SO_4_. The inset shows the zoomed-in image of D_2_ from 1:1 mixed acid and H_2_ from 100 mM D_2_SO_4_. The flux of each isotopic gas is shown in the *SI Appendix*, Fig. S6. (*C*) LSV recorded (ν = 5 mV s^−1^) during reduction of 1:1 acid mixture using an ECMS setup, with concurrent monitoring of HER, HDER, and DER.

As suggested by the ^17^O NMR results in Fig. 2*D*, increasing D_2_O content modifies the hydrogen-bonding environment of the bulk electrolyte. To further examine the dynamic consequence of this change, we carried out linear sweep voltammetry (LSV) on a Pt UME in 10 mM H_2_SO_4_ and 10 mM D_2_SO_4_. Because the steady-state current at a UME is directly related to mass transport under radial diffusion, the limiting current can be used to extract the diffusivity of protonic/deuteronic species. As shown in Fig. 3*A* and *SI Appendix*, Fig. S3, at approximately −1.0 V, before the transition into the water-reduction-dominated regime, the H_2_SO_4_ electrolyte exhibits a larger steady-state current than the D_2_SO_4_ electrolyte. The corresponding diffusion coefficients are $6.93\times 10^{-5}$ and $4.54\times 10^{-5}$ cm^2^ s^−1^ for H^+^ and D^+^, respectively. The resulting $D_{H^{+}}/D_{D^{+}}$ ratio of 1.53 is consistent with literature values and reflects the slower transport of deuterons relative to protons (65, 66). This slower H/D transport may also contribute to the reduced peak currents observed in the D_2_O-based ZnSO_4_ electrolyte despite the largely preserved Zn^2+^ charge-transfer kinetics shown in Fig. 2*B*. Overall, these measurements provide direct evidence that isotope substitution suppresses proton/deuteron hopping-mediated transport, a key component of solvent-mediated H/D reactivity.

We next examined whether slower deuteron dynamics translate into reduced gas evolution. ECMS was performed under galvanostatic conditions on a Cu working electrode at −0.2 mA in three acidic electrolytes: 100 mM H_2_SO_4_ in H_2_O, a 1:1 mixed acid containing 50 mM H_2_SO_4_ + 50 mM D_2_SO_4_ in 50/50 H_2_O/D_2_O, and 100 mM D_2_SO_4_ in D_2_O. In these systems, HER/HDER/DER kinetics are highly sensitive to the working electrode material. Cu was selected as a model substrate because it closely represents the current collectors commonly used in Zn battery anodes. ECMS enables real-time and highly sensitive quantification of gas-phase products. As shown in Fig. 3*B*, the total evolved gas amount, including H_2_, HD, and D_2_, decreases progressively with increasing deuterium content, from 230 nmol in 100 mM H_2_SO_4_ to 163 nmol in the mixed-acid electrolyte and 134 nmol in 100 mM D_2_SO_4_. This monotonic decrease likely arises from the combined effects of lower effective proton activity, slower D^+^ hopping dynamics, and isotope-dependent activation barriers for H/D transfer. Because these measurements were performed in the absence of Zn salt, the reduced gas evolution cannot be attributed to changes in Zn^2+^ solvation or Zn deposition kinetics. Instead, it reflects the intrinsic suppression of H/D evolution by deuterium substitution in a Zn-free electrolyte. Taken together, these results demonstrate that D_2_O itself suppresses solvent-mediated H/D reactivity through slower proton/deuteron transport and isotope-dependent reaction kinetics.

To complement the charge distribution obtained from galvanostatic measurements, LSV was coupled with operando ECMS to provide a potential-resolved view of isotopic gas evolution. Voltammetric measurements were conducted in the same three-electrode configuration at a scan rate of 5 mV s^-1^. Unlike chronopotentiometry (CP), LSV enables identification of the potentials at which individual gas-evolution channels are activated, as well as how their rates evolve with increasing cathodic driving force (67). As shown in Fig. 3*C*, the 50 mM H_2_SO_4_/D_2_SO_4_ mixed-acid electrolyte was examined. H_2_ and HD evolution initiate at similar potentials and increase rapidly as the voltage becomes more negative, whereas D_2_ formation requires a more negative potential and exhibits a more gradual increase in rate. This staggered isotopic onset and the different gas-evolution rates indicate that D_2_ evolution is kinetically less accessible than H_2_ or mixed H/D pathways. Such behavior reflects intrinsic kinetic differences associated with deuterium, where slower Grotthuss-type transport and larger zero-point-energy-related reorganization penalties increase the barrier for D-only reduction processes (50, 54). By jointly reducing H^+^/D^+^ transport and hydrogen evolution from the bulk electrolyte, deuterium substitution imposes a clear kinetic penalty on water-mediated reactivity. Such sluggish bulk dynamics are expected to suppress not only current-driven HER under cathodic polarization but also spontaneous processes governed by proton transfer and solvent reorganization. This bulk kinetic penalty therefore provides a direct mechanistic basis for reduced faradaic HER and mitigated resting loss, which will also be reflected in the ZnSO_4_ system discussed in the following section.

### 1.4. Zn-Coupled Gas Evolution Reveals Solvent-Isotope-Regulated HER Suppression.

Having established in Fig. 3 that deuterium substitution suppresses gas evolution in the absence of Zn, we next asked whether the same isotope-dependent kinetic penalty remains operative once Zn salt is introduced. This returns us to the practical ZnSO_4_ electrolyte, where Zn deposition and water reduction coexist at the same electrode. Our previous work showed that, on a Cu current collector, Zn plating and H_2_ evolution occur at similar onset potentials, indicating that parasitic hydrogen evolution is coupled to Zn electrodeposition rather than occurring as an isolated HER process (26). This overlap provides a platform in which the kinetic penalty of HER in D_2_O-based electrolytes becomes directly observable.

**Fig. 4.**
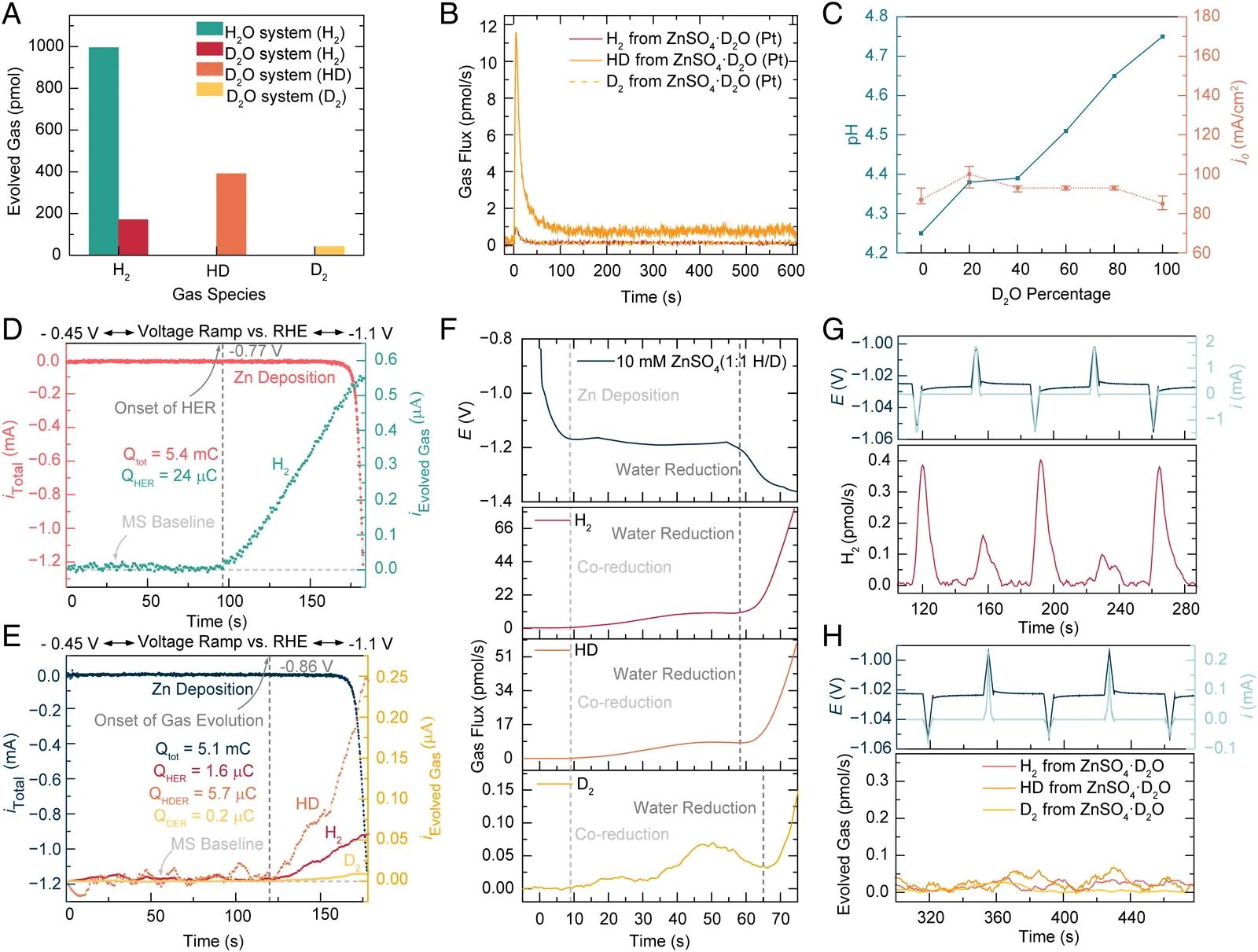
(*A*) Total evolved gas per deposition cycle (600 s) quantified by ECMS during galvanostatic polarization (-0.2 mA) in 1 M ZnSO_4_ electrolytes prepared with H_2_O and D_2_O. The flux is shown in *SI Appendix*, Fig. S11. (*B*) Isotope-resolved ECMS quantification of gas flux during ZnSO_4_ reduction in D_2_O-based electrolyte using a Pt working electrode under the same chronopotentiometric conditions. (*C*) Dependence of electrolyte pH and exchange current density (*j_0_*) on D_2_O fraction (0 to 100 vol%) in 1 M ZnSO_4_. LSV (ν = 5 mV s^−1^) recorded during zinc electrodeposition using an ECMS setup, with concurrent monitoring of (*D*) HER in the H_2_O-based system and (*E*) HER, HDER, and DER in the D_2_O based system. (*F*) In-situ ECMS measurement with galvanostatic deposition of zinc (-0.2 mA) in dilute (10 mM) 50/50 D_2_O/H_2_O-based ZnSO_4_ electrolyte. Small-potential-perturbation ECMS measurement in (*G*) H_2_O-based 1 M ZnSO_4_, (*H*) D_2_O-based 1 M ZnSO_4_, performed by cyclic voltammogram (ν = 5 mV s^−1^) ± 50 mV around *E_corr_*.

We first quantified the total gaseous products generated during galvanostatic Zn deposition by operando ECMS. As shown in Fig. 4*A*, under otherwise identical conditions, the H_2_O-based 1 M ZnSO_4_ electrolyte evolves 995 pmol of H_2_. In contrast, the D_2_O-based counterpart produces 171 pmol of H_2_, 391 pmol of HD, and 43 pmol of D_2_, corresponding to 605 pmol of total gas. Thus, D_2_O substitution reduces the total evolved gas from 995 to 605 pmol, corresponding to an approximately 39% reduction relative to H_2_O. This decrease is consistent with the acid-control results in Fig. 3*B*, where increasing deuterium content progressively lowers total gas evolution, and further indicates that isotope-dependent suppression of H/D evolution remains operative even in the presence of Zn^2+^ and active Zn plating. Moreover, the corresponding flux profiles (*SI Appendix*, Fig. S11) show that the H_2_ and HD signals in the D_2_O-based electrolyte reach quasi-steady values within the experimental window rather than decaying to zero. This sustained flux indicates that H-containing isotopologues are continuously replenished at the reactive interface, supporting the dynamic interfacial H/D balance proposed in Section 2.2 over the timescale of Zn deposition.

The isotopic distribution in the D_2_O-based ZnSO_4_ electrolyte is also mechanistically informative. Although D_2_O is the dominant solvent, HD remains the major gaseous product, followed by H_2_ and then D_2_. This distribution indicates that H-containing isotopologues remain available at the reactive interface, consistent with the mass-balance picture developed above in which ZnSO_4_‧7H_2_O introduces a substantial H-containing reservoir that is redistributed among H_2_O, HOD, and D_2_O. This dominance of HD reflects dynamic bulk–interface coupling: H/D exchange, transport of H-containing isotopologues, and interfacial Volmer–Heyrovsky-type pathways collectively determine the product distribution. Because D-only pathway remains kinetically less accessible, mixed H/D pathways can dominate even in a D-rich electrolyte (64, 68). To further test how the electrode surface affects isotopic selectivity, we performed the same galvanostatic ZnSO_4_/D_2_O ECMS measurement using Pt as the working electrode (Fig. 4*B*). Compared with Cu, the Pt electrode generates a substantially larger amount of D_2_, with 82 pmol D_2_ and 86 pmol H_2_, indicating nearly equal formation of D_2_ and H_2_. This increase in D_2_ is consistent with the stronger ability of Pt to catalyze adsorbed H/D recombination through a Tafel-type pathway. In a D_2_O-rich electrolyte, enhanced formation and recombination of adsorbed D on Pt can therefore increase D_2_ production relative to Cu. However, even on Pt, HD remains the dominant product, reaching 625 pmol. This observation shows that, despite the high D content of the solvent and the stronger catalytic ability of Pt toward surface recombination, the reactive interface is not converted into a purely D-derived environment. Instead, H-containing isotopologues are continuously dominated and participate in mixed H/D pathways. Thus, the Pt control supports the dynamic interfacial H/D picture: electrode identity can shift the relative contribution of Heyrovsky and Tafel steps, but the dominant HD product reveals persistent coupling between H-containing interfacial donors and the D-rich surrounding solvent network (63).

Moreover, we measured pH and *j*_0_ as a function of D_2_O fraction in 1 M ZnSO_4_ (Fig. 4*C*) to determine whether the isotope effect is accompanied by changes in Zn^2+^ electroreduction kinetics. With increasing D_2_O content, the electrolyte pH increases monotonically, consistent with reduced effective proton activity in the deuterated solvent network. In contrast, *j*_0_ remains nearly unchanged across the same solvent series, indicating that Zn^2+^ electroreduction kinetics are largely preserved within experimental resolution.

LSV-ECMS was then carried out to resolve the kinetics and onset behavior of isotopic gas evolution in both electrolytes. In the H_2_O-based electrolyte (Fig. 4*D*), HER begins at approximately -0.77 V versus RHE and increases rapidly upon further cathodic polarization. Integration of the gas signal yields a cumulative HER charge of 24 μC out of 5.4 mC applied. In contrast, the D_2_O-based electrolyte (Fig. 4*E*) exhibits a more negative onset of detectable gas evolution, around −0.86 V vs. RHE, together with much lower total gas-related charge consumption. Although isotopically distinct channels are observed in the D_2_O electrolyte, their combined cumulative charge is only 7.5 μC out of 5.1 mC applied. This reduction again shows that D_2_O suppresses water reduction in the ZnSO_4_ system. The approximately 0.1 V cathodic shift in gas-evolution onset from H_2_O to D_2_O is consistent with a slower H/D reduction process in the deuterated electrolyte, which can be attributed to lower effective proton activity, slower deuteron transport, and larger isotope-dependent reorganization barriers.

Another key observation, particularly for the D_2_O case in Fig. 4*E*, is that all gaseous products evolve at a common onset potential. This behavior is consistent with a common Zn-associated gating process and may include a corrosion-mediated contribution, wherein gas evolution is coupled to zinc plating on the Cu current collector. To understand this, let us step back and compare this to the acid system (Fig. 3*C*), where the staggered isotopic onset is not as apparent in the ZnSO_4_/D_2_O electrolyte. This contrast is mechanistically informative. In the acid-only system, where Zn is absent, the onset of each isotopic gas channel is governed more directly by the intrinsic reduction barrier of the corresponding H/D donor and by solvent reorganization in the interfacial water network. Under those conditions, the slower transfer and higher reorganization penalty associated with D-containing species make the D_2_ pathway less accessible, leading to its delayed onset relative to H_2_ and HD. This interpretation is broadly consistent with electrochemical isotope-effect studies showing that deuterium evolution is kinetically slower than hydrogen evolution and that the barrier difference can be expressed as a potential-dependent kinetic isotope effect (69). By contrast, in the ZnSO_4_ system, the nearly identical onset of H_2_, HD, and D_2_ suggests that gas formation is not controlled solely by the intrinsic isotope-dependent barrier of each individual channel. Rather, it is consistent with a common gating condition associated with the appearance of deposited Zn^0^ and the onset of Zn-coupled interfacial instability. In other words, once the interface enters the reducing regime where Zn electrodeposition occurs, Zn metal becomes available for spontaneous galvanic corrosion and for establishing the mixed-potential environment that supports hydrogen evolution on the electrochemically connected metallic network. Under this view, the ZnSO_4_ LSV-ECMS experiment is governed by two superimposed requirements. The first and primary requirement is the emergence of Zn^0^ metal itself, which enables Zn-coupled corrosion chemistry and creates a shared interfacial state from which all three isotopic gas channels can be released. This behavior is further examined in Fig. 4*G*. The secondary is the usual cathodic driving force needed to access hydrogen evolution. This is why the Zn system shows a common onset for H_2_, HD, and D_2_, whereas the acid-only system resolves the intrinsic isotope-dependent barriers more clearly. This distinction is further supported by Fig. 4*F*. More importantly, these observations suggest that suppressing solvent-mediated HER does not necessarily require sacrificing Zn^2+^ charge-transfer kinetics. Even in the ZnSO_4_ system, where Zn deposition and HER remain interfacially coupled, the parasitic H/D-evolution pathway can be weakened through regulation of the surrounding solvent network while Zn deposition kinetics are largely maintained. This finding is consistent with a growing body of literature demonstrating that bulk pH and proton transport properties of the electrolyte directly control the extent of HER during cycling and the magnitude of anode inventory loss during rest (33, 36).

To further distinguish Zn-coupled coreduction from direct water-reduction behavior, we performed galvanostatic zinc plating-ECMS in dilute 10 mM ZnSO_4_ electrolyte (50/50 H_2_O/D_2_O) (Fig. 4*F*). In this case, two distinct gas-evolution regimes are observed. At early times, around the onset of Zn reduction, H_2_, HD, and D_2_ appear together, consistent with Zn-coupled coreduction of H/D-containing water species. This behavior resembles the common-onset feature observed in concentrated ZnSO_4_ and indicates that, before Zn^2+^ transport limitation is reached, parasitic gas evolution is gated by Zn deposition and the associated interfacial/corrosion chemistry. At later times, around the transition in the voltage response, H_2_ and HD increase sharply while D_2_ shows a delayed increase. This second regime reflects a transition from Zn deposition-dominated coreduction to direct water reduction after depletion of Zn^2+^ near the electrode, analogous to Sand’s-time behavior under mass-transport limitation. Once Zn^2+^ transport can no longer sustain deposition, the electrode potential shifts into a water-reduction-dominated regime, and the intrinsic isotope-dependent kinetics reemerge; therefore, D_2_ formation becomes delayed relative to H_2_ and HD, similar to the staggered behavior observed in the Zn-free acid system.

Finally, we decoupled corrosion-mediated HER from cathodically driven HER and investigated the former near the corrosion potential (*E*_corr_) using small-potential-perturbation ECMS. A ±50 mV cyclic voltammetry was applied around *E*_corr_ in 1 M ZnSO_4_ while monitoring gaseous products in real time. In the H_2_O-based electrolyte (Fig. 4*G*), H_2_ evolution is observed during both plating and stripping. This behavior is consistent with a corrosion-mediated mechanism in which both cathodic and anodic perturbations renew or expose fresh Zn surface sites, increasing the fraction of electrons diverted through Zn corrosion coupled to HER. During plating, newly deposited Zn provides reactive metallic sites that can undergo spontaneous galvanic corrosion. During stripping, partial dissolution and surface restructuring can also expose fresh, less passivated Zn, allowing corrosion-mediated HER to persist even under anodic polarization. This interpretation is consistent with a mixed-potential framework in which HER near *E*_corr_ reflects modulation of an underlying corrosion process rather than the initiation of a completely independent faradaic HER pathway. Under the same ±50 mV perturbation in the D_2_O-based electrolyte (Fig. 4*H*), H_2_, HD, and D_2_ remain below the ECMS detection limit. This strong suppression of corrosion-associated gas evolution, together with the similar *E*_corr_ compared with the H_2_O-based electrolyte, supports two central aspects of the isotope-substitution platform. First, the D-rich bulk solvent network strongly suppresses corrosion-mediated H/D evolution by slowing H/D transport and solvent reorganization. Second, the H-containing isotopologues introduced by ZnSO_4_‧7H_2_O help buffer the Zn^2+^-centered interfacial environment, preserving similar solvation energetics and corrosion potential. Thus, even though Zn deposition, corrosion, and H/D evolution remain dynamically coupled, D_2_O substitution selectively weakens the parasitic gas-evolution pathway while maintaining Zn^2+^-centered electrochemical behavior.

Together, the ECMS, FSCV, LSV, and small-perturbation measurements reveal two mechanistic features of the hydrated ZnSO_4_/D_2_O platform. First, Zn deposition gates the onset of parasitic gas evolution: H_2_, HD, and D_2_ appear together with Zn deposition. Second, the D-rich solvent network suppresses the extent of both faradaic and corrosion-mediated gas evolution without substantially altering Zn^2+^ charge-transfer kinetics or *E*_corr_. These results support a dynamic coupling picture in which H-containing isotopologues remain available near the Zn^2+^-centered interface, but their continued consumption is limited by H/D exchange, transport, and solvent reorganization within the surrounding D-rich bulk. These findings challenge the prevailing paradigm that solvation shell modulation is the primary route to enhanced Zn stability, since such approaches can also introduce a kinetic penalty for Zn plating by altering Zn^2+^ desolvation and charge-transfer behavior. Instead, our results show that parasitic H/D evolution can be suppressed by regulating the surrounding solvent network and diffuse-layer H/D activity while largely preserving Zn^2+^ electroreduction kinetics. This distinction also provides a useful perspective for interpreting additive effects: Additives may improve stability not only by modifying bulk solvation structure but also by preferentially adsorbing at the interface, reorganizing the EDL, and regulating local H/D donor accessibility near the electrode (70–72).

### 1.5. Solvent-Network Suppression Translates into Enhanced Zn Cycling Reversibility and Mitigated Resting Loss.

Having established that D_2_O-rich solvent networks suppress H/D evolution while largely preserving Zn^2+^ electroreduction kinetics, we next examined whether this mechanistic effect translates into improved Zn metal anode reversibility. The isotope-resolved ECMS measurements above show that D_2_O substitution attenuates both faradaic and corrosion-mediated gas evolution. Therefore, in this section, we connect the mechanistic picture developed above with the electrochemical, structural, and morphological consequences observed at the cell level.

**Fig. 5.**
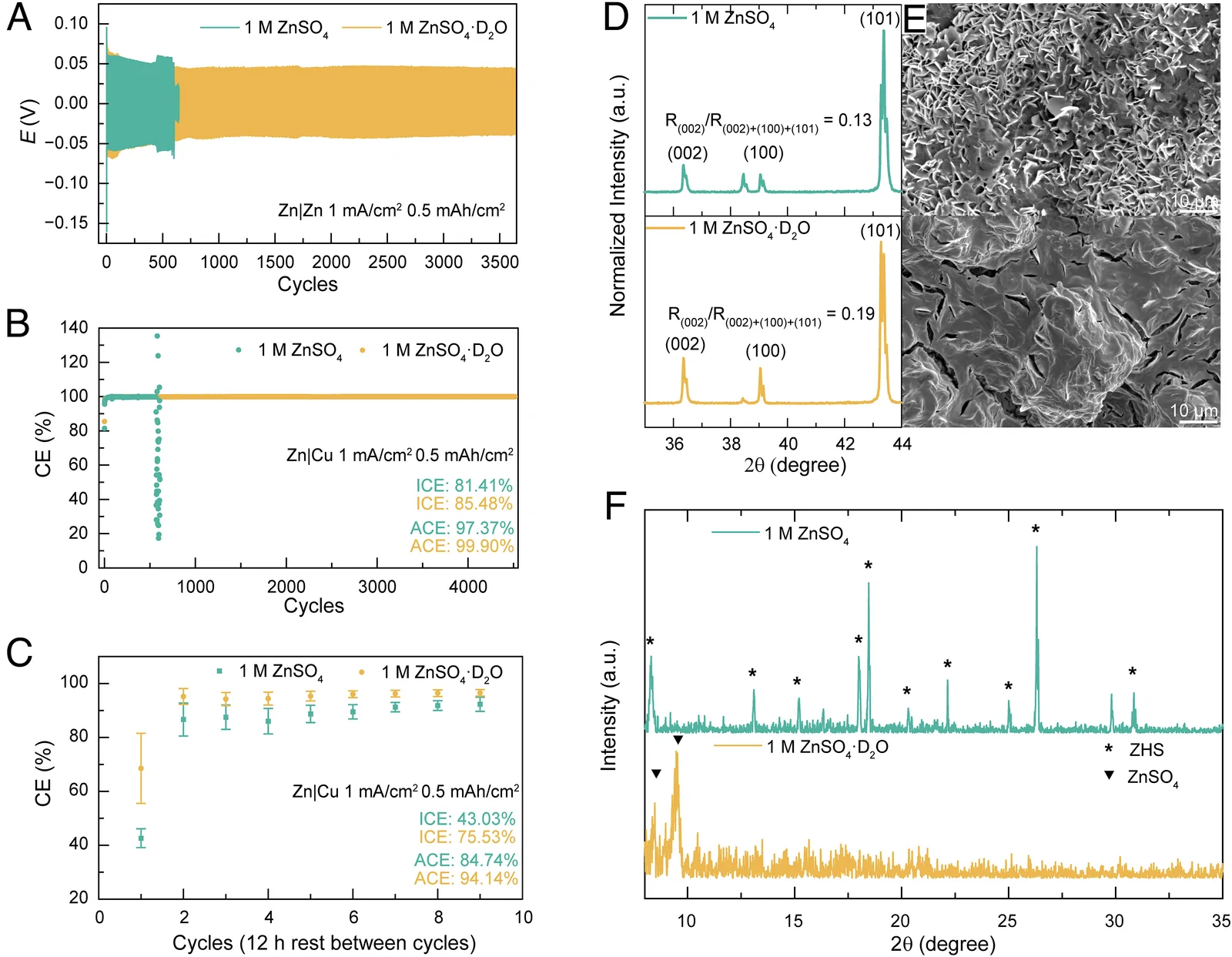
(*A*) Zn||Zn symmetric cell cycling at a current density of 1 mA cm^−2^ and a capacity of 0.5 mAh cm^−2^ in 1 M ZnSO_4_, comparing electrolyte systems with H_2_O and D_2_O as solvent. (*B*) CE of Cu||Zn asymmetric cells cycled at a current density of 1 mA cm^−2^ and a capacity of 0.5 mAh cm^−2^ in 1 M ZnSO_4_, evaluated in H_2_O- and D_2_O-based electrolytes. (*C*) Zn||Cu cycling performance with a 12 h rest inserted between cycles. (*D*) Normalized XRD patterns of electrodeposited samples with deposition angles between 35 to 44° at 1 mA cm^−^^2^ and 0.5 mAh cm^−^^2^, characterizing the SEI morphology within different solvent. (*E*) SEM images of zinc plating on Cu foil within H_2_O and D_2_O at 1 mA cm^−^^2^ and 0.5 mAh cm^−^^2^. (Scale bar: 10 µm) (*F*) Normalized XRD patterns of electrodeposited samples over 2θ = 8-35° at 1 mA cm^−^^2^ and 0.5 mAh cm^−^^2^, corresponding to side-product formation in H_2_O- and D_2_O-based electrolytes.

We first evaluated Zn plating/stripping stability in Zn||Zn symmetric cells using 1 M ZnSO_4_ prepared with either H_2_O or D_2_O. As shown in Fig. 5*A*, the D_2_O-based electrolyte sustains stable cycling for over 3,600 h at 1 mA cm^−2^ and 0.5 mAh cm^−2^, representing an improvement of approximately 3,000 h over the H_2_O-based electrolyte. A similar trend is observed in Zn||Cu asymmetric cells. Under the same testing conditions, the D_2_O-based system delivers 4,500 cycles with a constant CE of 99.90%, whereas the H_2_O-based electrolyte fails after only 600 cycles with a sharp drop in CE (Fig. 5*B*). The markedly prolonged cycling stability and higher reversibility in the D_2_O system again point to reduced parasitic Zn consumption. Moreover, as shown in *SI Appendix*, Fig. S15, the similar nucleation overpotentials and polarization voltages observed in symmetric cells for D_2_O and H_2_O electrolytes confirm that interfacial properties are effectively preserved. To more directly examine the role of resting loss, Zn||Cu cells were further tested with a 12 h rest inserted between cycles. As shown in Fig. 5*C*, both the initial CE (ICE) and average CE (ACE) are substantially lower than those obtained under continuous cycling, confirming that idle-time Zn loss is indeed a major contributor to inefficiency in aqueous Zn systems. Even under this deliberate harsh condition, the D_2_O-based electrolyte still improves ICE by about 30% and ACE by about 10% relative to the H_2_O system. This result is fully consistent with the mechanistic picture developed in the previous sections that D-rich solvent-network dynamics can mitigate not only current-driven H/D evolution during plating but also spontaneous Zn loss during rest.

Next, we correlated the electrochemical performance with morphological and structural characteristics by performing XRD and SEM analyses. Fig. 5*D* displays XRD patterns of Zn deposits within the 2θ region of 35-44° in both electrolyte systems. From the literature, Zn crystallographic orientations of (100) and (101) are typically associated with dendritic or intermediate growth modes, whereas the low-index (002) orientation corresponds to compact, planar deposition that resists corrosion and promotes uniformity. To quantitatively evaluate morphological differences, we compared the relative intensity ratio *R*_002_/(*R*_100_)+(*R*_101_)+(*R*_002_) for deposits obtained from H_2_O- and D_2_O-based electrolytes. Notably, the D_2_O-based electrolyte exhibited a higher intensity ratio (0.19), indicating a preference for the (002) orientation and thus more uniform and planar Zn deposition, which is responsible to the longer cycling of coin cells. SEM images in Fig. 5*E* further underscore these differences. Zn plated in H_2_O exhibited disordered, porous morphology with evidence of byproduct agglomeration. This morphology is consistent with dendrite-prone growth and Zn(SO_4_)_x_(OH)_y_ (ZHS) accumulation. In contrast, the D_2_O-deposited Zn appeared smooth and compact, with significantly fewer surface artifacts. Notably, the diffraction feature at ~38.5° is from the Cu substrate and is weaker in the D_2_O-based electrolyte (49). This attenuation is likely associated with improved Zn deposition that more effectively covers the Cu substrate, thereby suppressing substrate peaks. Consistent with this interpretation, the absence of hexagonal flakes and particulate debris in the D_2_O-deposited Zn further suggests effective suppression of corrosion-related byproducts (73–78).

To further confirm the suppression of side reactions, we examined the XRD patterns within the 8-35° region (Fig. 5*F*). Characteristic peaks associated with ZHS, and other basic Zn salts were prominent in the H_2_O electrolyte, while nearly absent in the D_2_O system. The presence of only a few residual peaks around 9° in the D_2_O-based electrolyte corresponds to ZnSO_4_ crystallites, which further confirms minimized side product accumulation. Altogether, these results demonstrate that regulating the bulk H/D solvent network translates directly into practical gains in Zn anode stability. By mitigating both faradaic HER during cycling and spontaneous Zn loss during rest, D_2_O reduces OH^-^-driven byproduct formation, promotes denser and more strongly (002)-oriented Zn deposition, and substantially extends cell lifetime (79–82). These results complete the central picture of this study: parasitic side reactions can be suppressed through control of solvent-network transport and reorganization, without requiring a major kinetic penalty to Zn interfacial charge transfer.

### 1.6. Reconciling Solvation, Interfacial Feedback, and Bulk Transport as Distinct HER Control Levels.

The results presented here provide a framework to reconcile the present isotope-substitution study with our previous mechanistic descriptions of HER in aqueous Zn batteries. Our earlier work on the kinetic origin of HER addressed the molecular origin and onset of hydrogen evolution during Zn deposition. In that study, Zn deposition and H_2_ evolution were observed to emerge at nearly the same onset potential, consistent with the participation of Zn^2+^-coordinated water during electrodeposition. This result led us to view Zn deposition as a kinetic “gate” for coevolving HER in two related ways. First, corrosion-mediated HER requires the formation of metallic Zn. Once Zn nuclei form on the current collector, Zn^0^ can spontaneously oxidize, and the released electrons can drive coupled HER on electronically connected surfaces. Second, for faradaic coreduction, water molecules associated with the Zn^2+^ solvation environment become electrochemically relevant as Zn^2+^ approaches the interface and undergoes desolvation/electroreduction. Because Zn^2+^-coordinated water is more acidic than bulk water, the potential window for Zn electroreduction can also activate proton-coupled water reduction. Therefore, even if Zn deposition and HER proceed through distinct elementary pathways, the onset of Zn deposition can still govern when and where HER becomes activated (26). Importantly, the hydrated ZnSO_4_/D_2_O platform provides a uniquely stringent mechanistic test of Zn-mediated corrosion and Zn-gated HER. By simultaneously monitoring H_2_, HD, and D_2_, this platform resolves multiple isotopic gas products that are not accessible in conventional H_2_O-based aqueous systems, including our previous “kinetic origin” work where only H_2_ evolution could be monitored. The isotope-resolved ECMS results show that all gaseous products emerge concurrently with Zn deposition, providing stronger support for a common Zn-mediated interfacial/corrosion pathway rather than independent HER/HDER/DER pathways with distinct activation behavior. In other words, the shared onset of H_2_, HD, and D_2_ indicates that parasitic gas evolution is activated by the appearance of Zn and the associated mixed-potential/corrosion environment, while the isotopic solvent network controls how extensively gas evolution proceeds.

This interpretation also explains why many additive and cosolvent strategies that suppress HER often simultaneously alter Zn^2+^ desolvation barriers, Zn^2+^ charge-transfer behavior, or solvation-shell structure. For example, in our previous “electrolyte-controlled hydrogen-evolution” work, modulation of Zn electrodeposition kinetics was shown to either improve or compromise anode stability depending on how strongly Zn deposition *j*_0_ is slowed. Moderate slowing of Zn deposition kinetics can promote favorable nucleation/growth and suppress HER, whereas excessive slowing shifts charge consumption toward corrosion-dominated pathways and worsens side reactions (49). Thus, in additive/cosolvent systems, HER suppression is frequently coupled to reduced water participation in the Zn^2+^ solvation shell, weakened solvation-shell hydrogen bonding, or increased nucleation/desolvation barriers, which can either deliberately or unintentionally delay the Zn-gated onset of water reduction.

The present work highlights another critical component: H^+^/H-containing donor mass transport. Mildly acidic Zn electrolytes typically have pH values near 4, corresponding to an equilibrium H^+^ concentration of ~0.1 mM. Although this concentration is small compared with bulk water, proton donors are continuously consumed during HER and corrosion-mediated reactions at the electrode surface. Therefore, the rate at which H^+^, HOD, H_2_O, and other H-containing donors are replenished through the diffuse layer can strongly influence the sustained HER flux. In the hydrated ZnSO_4_/D_2_O electrolyte, crystal-water-derived H-containing isotopologues help maintain an H-buffered Zn^2+^ solvation and near-interfacial environment, whereas the surrounding D-rich bulk limits both the amount and the rate of H-containing donor supply. At the same time, sluggish deuteron and D-containing donor transfer kinetics and slower D-rich solvent reorganization further suppress the overall side-reaction rate. This interpretation connects directly to our interfacial pH-gradient framework. In that work, at high current density, HER during Zn plating generated OH^–^ near the electrode faster than the surrounding electrolyte could neutralize the interface or replenish the local proton-donor environment. This reaction-transport imbalance produced an interfacial pH gradient, increased the local HER overpotential, and promoted protective interphase formation under suitable current-density regimes. Therefore, the pH-gradient effect can be viewed as the interfacial manifestation of limited proton-donor transport during ongoing HER. The present isotope-substitution study tunes the same balance from the solvent side: the D-rich bulk/diffuse layer slows H/D donor transport, hydrogen-bond rearrangement, and solvent reorganization before those donors reach the reactive interface. As a result, both faradaic HER during Zn deposition and corrosion-mediated gas evolution during rest are suppressed (53).

Taken together, these results provide an integrated framework in which Zn deposition gates the onset of parasitic gas evolution, Zn^2+^ electroreduction kinetics and interfacial feedback regulate current partitioning during plating, and proton and H-containing donor transport through the bulk/diffuse layer controls the sustained HER/HDER/DER flux.

## 2. Conclusion

In this work, we demonstrate that solvent isotope substitution provides a mechanistic route to suppress parasitic H/D evolution in aqueous ZnSO_4_ electrolytes while largely preserving Zn^2+^ electroreduction kinetics. Through combined spectroscopic, electroanalytical, and operando ECMS measurements, we show that the hydrated ZnSO_4_‧7H_2_O/D_2_O system maintains a similar Zn^2+^-centered coordination environment and interfacial electrochemical response within experimental resolution, as reflected by minimal changes in ^67^Zn NMR, *j_0_*, C_dl_, OCP, crossover potentials, and Zn^2+^ transport. In contrast, D_2_O substitution strongly perturbs the surrounding H/D solvent network, reducing effective proton activity, slowing H/D transport, and increasing solvent-reorganization barriers. These solvent-network effects suppress both faradaic H/D evolution during Zn deposition and corrosion-mediated gas evolution during rest, leading to reduced side product formation, denser Zn growth, improved resting-loss-sensitive CE, and markedly enhanced cycling reversibility. The isotope-resolved ECMS results further show that Zn deposition gates the onset of parasitic gas evolution, while the D-rich bulk controls the flux of H/D-containing donors that sustains HER/HDER/DER. Unlike conventional H_2_O-based aqueous systems, where only H_2_ evolution is typically resolved, this platform enables simultaneous detection of H_2_, HD, and D_2_. The concurrent emergence of all three isotopic gas products with Zn deposition provides strong support for a common Zn-mediated interfacial/corrosion pathway, rather than independent HER/HDER/DER pathways with distinct activation behavior. Together with our prior studies, these findings establish HER in aqueous Zn batteries as a regime-dependent process governed by coupled Zn^2+^ solvation/desolvation, interfacial pH/EDL/interphase evolution, corrosion dynamics, and bulk solvent-network transport. More broadly, this work identifies bulk and diffuse-layer water dynamics as a critical design variable for aqueous Zn metal batteries. Rather than relying solely on solvation-shell reconstruction, interfacial additives, or increased Zn^2+^ desolvation barriers, parasitic gas evolution can also be suppressed by regulating solvent-network transport and reorganization while maintaining favorable Zn deposition kinetics. Future studies using interface-sensitive operando techniques, such as in situ Raman spectroscopy and infrared spectroscopy, could further resolve dynamic changes in interfacial water structure, H/D isotopologue distribution, local pH/pD, and Zn^2+^ solvation during electrodeposition, providing deeper insight into Zn plating coupled with parasitic side reactions.

## 3. Materials and Methods

*SI Appendix* provides detailed descriptions of the materials and reagents used, electrolyte preparation, ultramicroelectrode fabrication and validation, and experimental characterization techniques. It further outlines the electrochemical measurement procedures, Tafel analysis methodology, and FSCV approach for determining Zn^2+^ diffusion coefficients. Comprehensive details of the operando ECMS setup, calibration procedures for H_2_, D_2_, and HD detection, and data analysis protocols are also included. These methodological details ensure the reproducibility and validation of the experimental results presented in the main text.

## Supplementary Material

Appendix 01 (PDF)

## Data Availability

All study data are included in the article and/or *SI Appendix*.
