# Supplementary material for: Decoupling proton reactivity from Zn2+ interfacial electrochemistry through solvent isotope substitution
Source: Proc Natl Acad Sci U S A. 2026 Sep 8;123(37):e2619771123. doi: 10.1073/pnas.2619771123 (PMC13578406; doi:10.1073/pnas.2619771123)
Supplement: Supplementary file 1 [file pnas.2619771123.sapp.pdf]

Supplementary Information

Decoupling proton reactivity from Zn^{2+} interfacial electrochemistry through solvent isotope substitution

Taizhe Liu[a, †], Md. Arif Faisal[a, †], Ashutosh Rana[a, †], Ashutosh Bhadouria[a,c], James H. Nguyen[a], Saptarshi Paul[a], Brian M. Tackett[a,c] and Jeffrey E. Dick[a,b]*

[a] Department of Chemistry, Purdue University, West Lafayette, IN, 47907, USA
[b] Elmore Family School of Electrical and Computer Engineering, Purdue University, West Lafayette, IN, 47907, USA
[c] Davidson School of Chemical Engineering, Purdue University, West Lafayette, IN, 47907, USA

† These authors contributed equally

Corresponding Author:
Jeffrey E. Dick (jdick@purdue.edu)

Experimental Section

1. Materials

Reagent-grade zinc sulfate heptahydrate ($ZnSO_4 \cdot 7H_2O$), hexaammineruthenium(III) chloride, reagent-grade potassium chloride (KCl), sulfuric acid (H_2SO_4), Sulfuric acid-d2 (D_2SO_4) and deuterium oxide (99.9 atom %D) were purchased from Sigma-Aldrich. Zinc and copper foils (99.99% purity) were acquired from MTI Supplies, while 25 μm tungsten and copper metal wires were procured from Goodfellow. All chemicals were used as received without additional purification. Deionized (DI) water for aqueous electrolyte preparation was obtained from a Milli-Q ultrapure water system.

2. Preparation of the Electrolyte

The electrolytes were prepared by dissolving 1 mol/L (M) $ZnSO_4$ in deionized (DI) water/ deuterium oxide (D_2O) at room temperature (25 °C).

3. Method of Testing the W Ultramicroelectrode

A two-electrode configuration was employed for cyclic voltammetry (CV) measurements involving a homemade tungsten ultramicroelectrode (W UME) as the working electrode. The small magnitude of current passed during measurements significantly reduces the potential drop at the working electrode, thus ensuring reliable referencing against the stable potential of the Ag/AgCl (1 M KCl) reference/counter electrode. All electrochemical analyses were conducted using 1 M $ZnSO_4$ as the supporting electrolyte. Prior to initiating zinc electrodeposition on the tungsten UME, cyclic voltammetry was performed with hexaammineruthenium(III) chloride (RHT), an outer-sphere redox probe, to verify the presence of steady-state current behavior characteristic of ultramicroelectrodes. A representative cyclic voltammogram, displaying the distinctive sigmoidal shape typical of UMEs, was obtained under identical experimental conditions utilizing 5 mM RHT and is presented in ***Fig. S16***. The observed steady-state currents coupled with the low capacitive background confirm the successful fabrication and functionality of the W UME.(1–3)

4. Characterization

Zinc foils were converted into discs and used as Zn electrodes for symmetric cell, whereas Copper discs served as Cu electrodes for asymmetric cell. Zinc electrodeposition on copper foils was performed for X-ray diffraction (XRD) analysis. Morphological characterization was conducted using field emission scanning electron microscopy (FESEM, JEOL JSM-7500F). Phase structure exploration was performed by XRD (Bruker D8 advanced) with Cu Kα radiation over the 0° to 45° 2 theta range. Zn nuclear magnetic resonance (^{67}Zn NMR) and proton NMR (^{1}H NMR) were carried out by Bruker NMR (500 MHz and 300 MHz respectively). Fourier-transform infrared spectroscopy (FTIR) was achieved by Attenuated Total Reflectance Fourier transform infrared spectroscopy (Thermo-Nicolet 6700 Series).

5. Electrochemical Measurements

All electrochemical measurements were conducted using a CHI6284E electrochemical workstation. As detailed in the main text, a two-electrode configuration was employed for the CV measurements.

6. Equations for Tafel analysis(4)

$$\textbf{Cathodic Reaction: } Log|i| = Log(i_0) - \left(\frac{\alpha F}{2.3RT}\right)\eta$$

$$\textbf{Anodic Reaction: } Log|i| = Log(i_0) + \left(\frac{(1-\alpha)F}{2.3RT}\right)\eta.$$

Where i is current, i_0 is exchange current, η is overpotential, α is the transfer coefficient, F is Faraday's constant, T is the temperature in Kelvins, R is universal gas constant.

7. Details of Fast Scan Cyclic Voltammograms and Measurement of Diffusion Coefficient

The diffusion coefficient in 1 M $ZnSO_4$ and 1 M $ZnSO_4 \cdot D_2O$ is represented in ***Fig. 1(c)***, using the peak observed during the forward sweep of the FSCVs. The equation of peak current as a function of scan rate for irreversible redox reaction,

$$j_p = (2.99 \times 10^5)\, n^{\frac{3}{2}}\, C_{Zn^{2+}}\, \alpha^{\frac{1}{2}} D_{Zn^{2+}}^{\frac{1}{2}}\, \nu^{\frac{1}{2}}$$

was used to determine the diffusion coefficient using the slope of the fit between j_p and $\nu^{0.5}$. Where, j_p is the peak current density (peak current divided by geometric area), n is the number of electrons transferred, $C_{Zn^{2+}}$ is the concentration of zinc ions, α is the transfer coefficient, $D_{Zn^{2+}}$ is the diffusion coefficient of Zn^{2+} and ν is the scan rate.(4)

8. ECMS

Electrochemical mass spectrometry (ECMS, Spectro Inlets ApS, Copenhagen) was employed to monitor volatile products evolved during electrodeposition in real time. The system integrates a membrane-based electrochemical cell with a mass spectrometer, enabling quantitative gas detection with high sensitivity and isotope resolution. In the cell design, a circular Cu disk working electrode (active area ≈ 0.196 cm^2) was positioned approximately 100 μm from a semipermeable membrane chip, separated by a thin electrolyte layer. The membrane chip was continuously purged with research-grade helium (6N, A-OX Welding), supplied through an internal mass flow controller. This helium stream maintained a stable pressure differential across the membrane while efficiently transporting volatile species to the MS inlet. The short diffusion distance between the working electrode and the membrane ensured near 100% collection efficiency of gaseous products, while narrow channels within a Kel-F block preserved ionic conductivity across the thin electrolyte layer and minimized bulk species crossover.

Electrochemical measurements were carried out in a three-electrode configuration. The Cu disk served as the working electrode, an Ag/AgCl reference electrode was immersed in 3.4 M KCl

solution (eDAQ), and a platinum wire was employed as the counter electrode. All electrodes were connected to a Biologic SP-300 potentiostat controlled by EC-Lab software. The ECMS system was operated using Zilien software (Spectro Inlets), which provided integrated control of valves, carrier gas flow, and synchronization of MS data acquisition with electrochemical protocols.

Following data collection, ECMS datasets were processed using the ixdat Python package, a framework specifically developed for ECMS analysis. This allowed systematic treatment of time-resolved mass spectrometric signals, accurate quantification of product formation rates, and direct correlation with the electrochemical response.(5)

The chronopotentiometry (CP) for acid reduction and Zn electrodeposition on ECMS is shown in ***Fig. S4*** and ***Fig. S8.***

Calibration: Operando detection of hydrogen evolution during Zn deposition was achieved using internal calibration standards. A polycrystalline Pt disk electrode (5.0 mm OD × 4.0 mm height, mirror-polished; Pine Research Instrumentation) was immersed in 1 M $HClO_4$ and subjected to CP to generate steady-state hydrogen evolution (HER). A sequence of CP holds was applied in both increasing (ramp-up) and decreasing (ramp-down) current steps. The mass spectrometry signal corresponding to H_2 (m/z = 2) was integrated and correlated with the theoretical amount of H_2 evolved, calculated from Faraday's Law under the assumption of 100% Faradaic efficiency-justified for Pt under acidic conditions in the ECMS setup. The resulting calibration curve, shown in ***Fig. S13***, yields a calibration factor of 0.456 C/mol, which was subsequently used for all HER flux quantification. Further methodological details are available in prior literature.(6) To extract the H_2 flux (pmol/s), the MS signal was divided by this calibration factor. This flux was then converted to charge or current via Faraday's Law.
To quantify isotopic hydrogen evolution during Zn electrodeposition, calibration factors for D_2 were established using a predictive theory. It was estimated based on ionization cross-section theory described by Scott ***et al.***, where:

$$\boldsymbol{f_i = k \cdot \sigma_i \cdot \frac{I_M^i}{\sum_M I_M^i} \cdot T(M)}$$

Here, $\boldsymbol{\sigma_i}$ is the ionization cross-section of D_2 at 70 eV, $\boldsymbol{I_M^i}$ is the intensity at mass-to-charge (m/z) = 4, and $\boldsymbol{T(M)}$ is the transmission function. Applying this method yielded a predicted calibration factor of 0.391 C/mol.(7)
To experimentally determine the calibration factor for HD, we employed a differential current analysis during stepped chronoamperometry in a 1 M D_2SO_4/D_2O electrolyte. Assuming HER, HDER, and DER all proceed via two-electron pathways and that H/D scrambling in the ion source is negligible, the current contributions were deconvoluted as:

$$\boldsymbol{i}_{\text{total}} = \boldsymbol{i}_{\text{HER}} + \boldsymbol{i}_{\text{DER}} + \boldsymbol{i}_{\text{(H–D)ER}}$$

Where $\boldsymbol{i}_{\text{HER}}$ and $\boldsymbol{i}_{\text{DER}}$ were calculated from their respective MS signals using known calibration factors for H_2 and D_2. The residual current was attributed to HD formation ($\boldsymbol{i}_{\text{(H–D)ER}}$), and a calibration curve was constructed by plotting the integrated MS signal at m/z = 3 against the calculated $\boldsymbol{i}_{\text{(H–D)ER}}$. This yielded a calibration factor of 0.0715 C/mol, with a strong linear

correlation, shown in ***Fig. S14***. These factors were then used to convert raw MS signals into real-time isotopic hydrogen fluxes throughout the study.

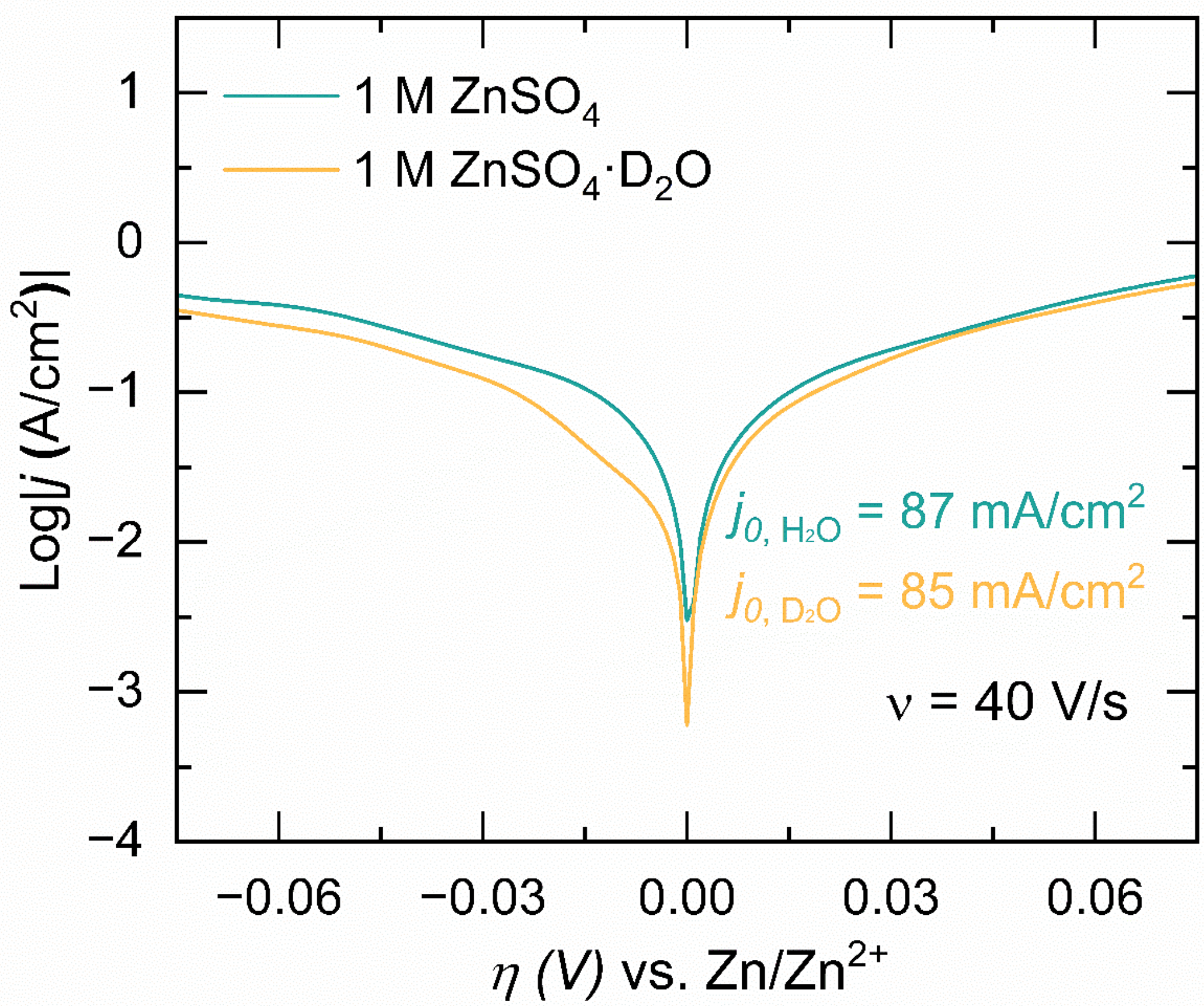

Fig S1. Overlay of Tafel plot that is derived from backward sweep of the cyclic voltammogram at scan rate of 40 V/s within kinetic regime.

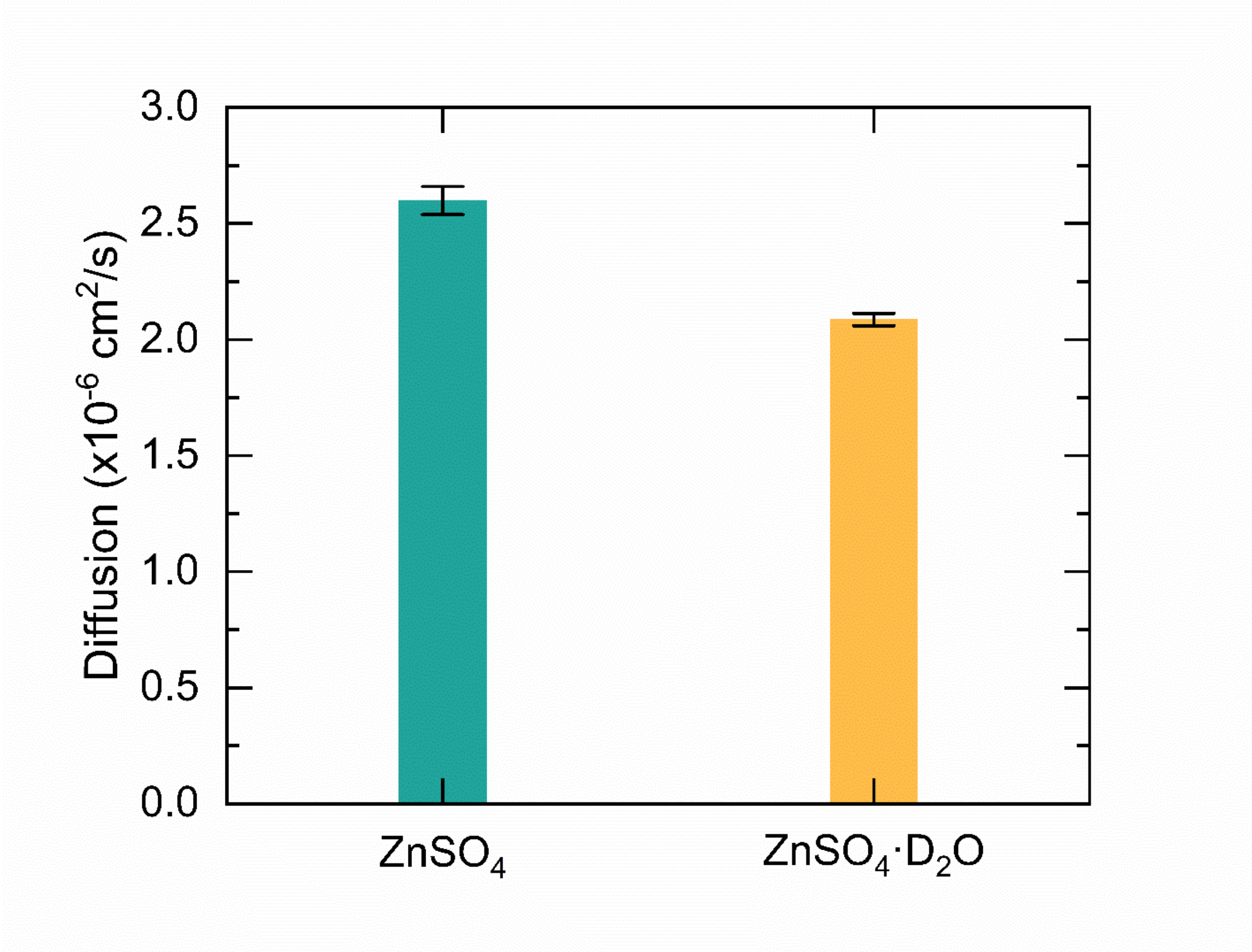

Fig S2. Diffusion coefficient ($D_{Zn^{2+}}$) of Zn^{2+} in H_2O and D_2O in 1 M $ZnSO_4$ with error bars.

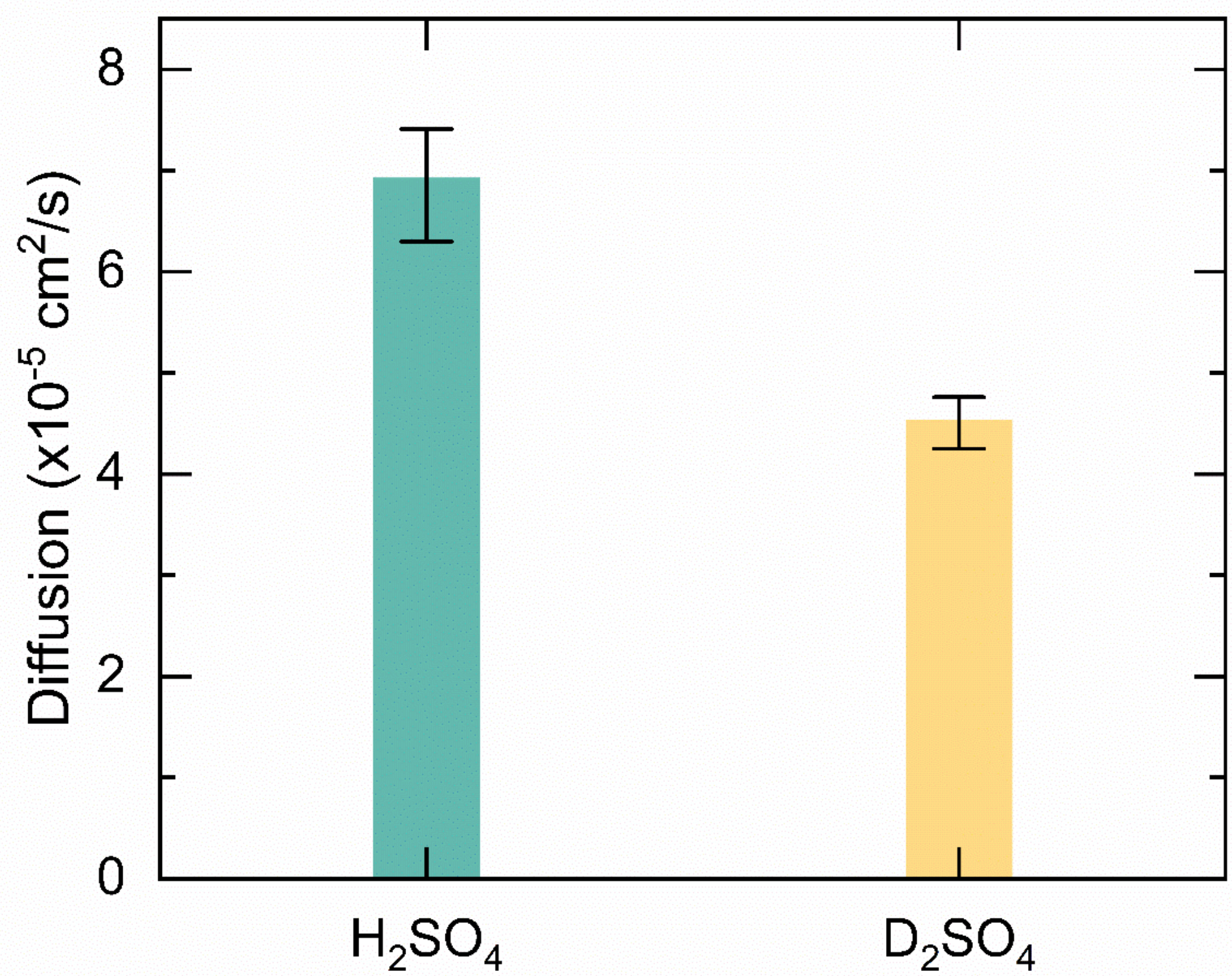

Fig S3. Proton and deuteron diffusion coefficients (D_{H^+} and D_{D^+}) of 10 mM H_2SO_4 and 10 mM D_2SO_4 with error bar.

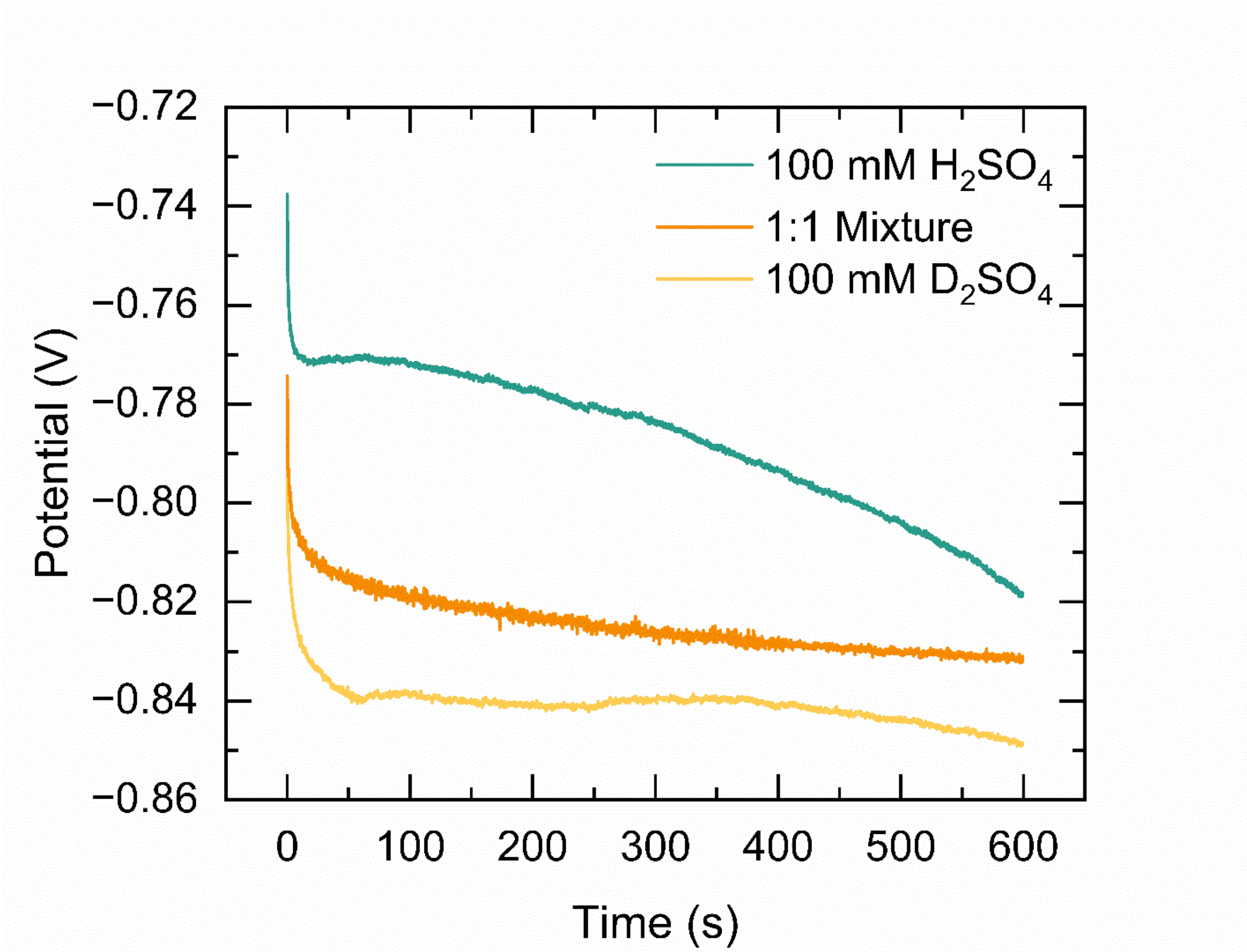

Fig. S4. Chronopotentiometry (CP) at -0.2 mA recorded using ECMS setup in a 3-electrode setup showing the real-time acid reduction.

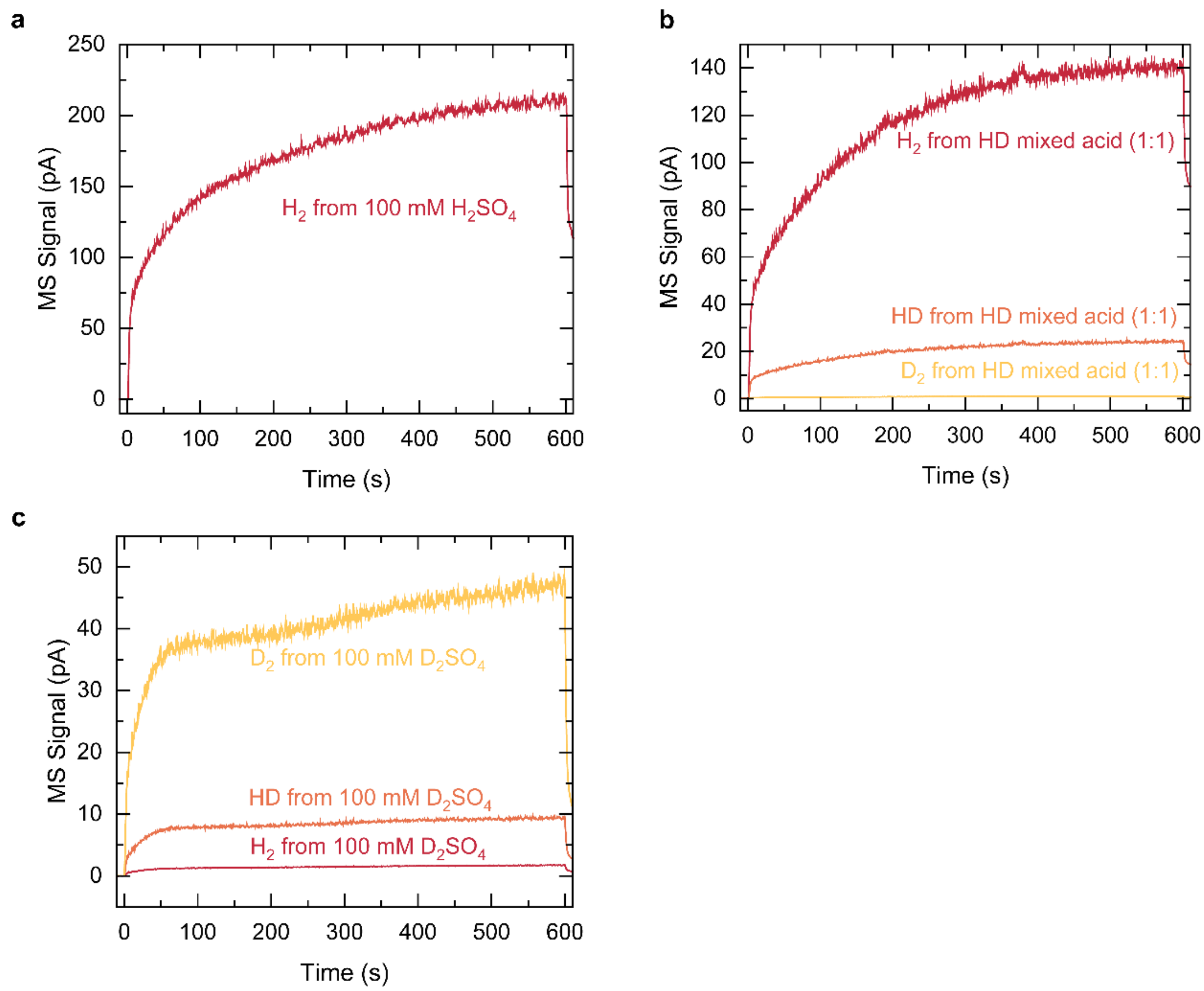

Fig. S5. Raw mass spectrometry (MS) data collected during CP at -0.2 mA in **(a)** 100 mM H_2SO_4, **(b)** 1:1 mixed H/D acid (50 mM H_2SO_4+ 50 mM D_2SO_4), and **(c)** 100 mM D_2SO_4.

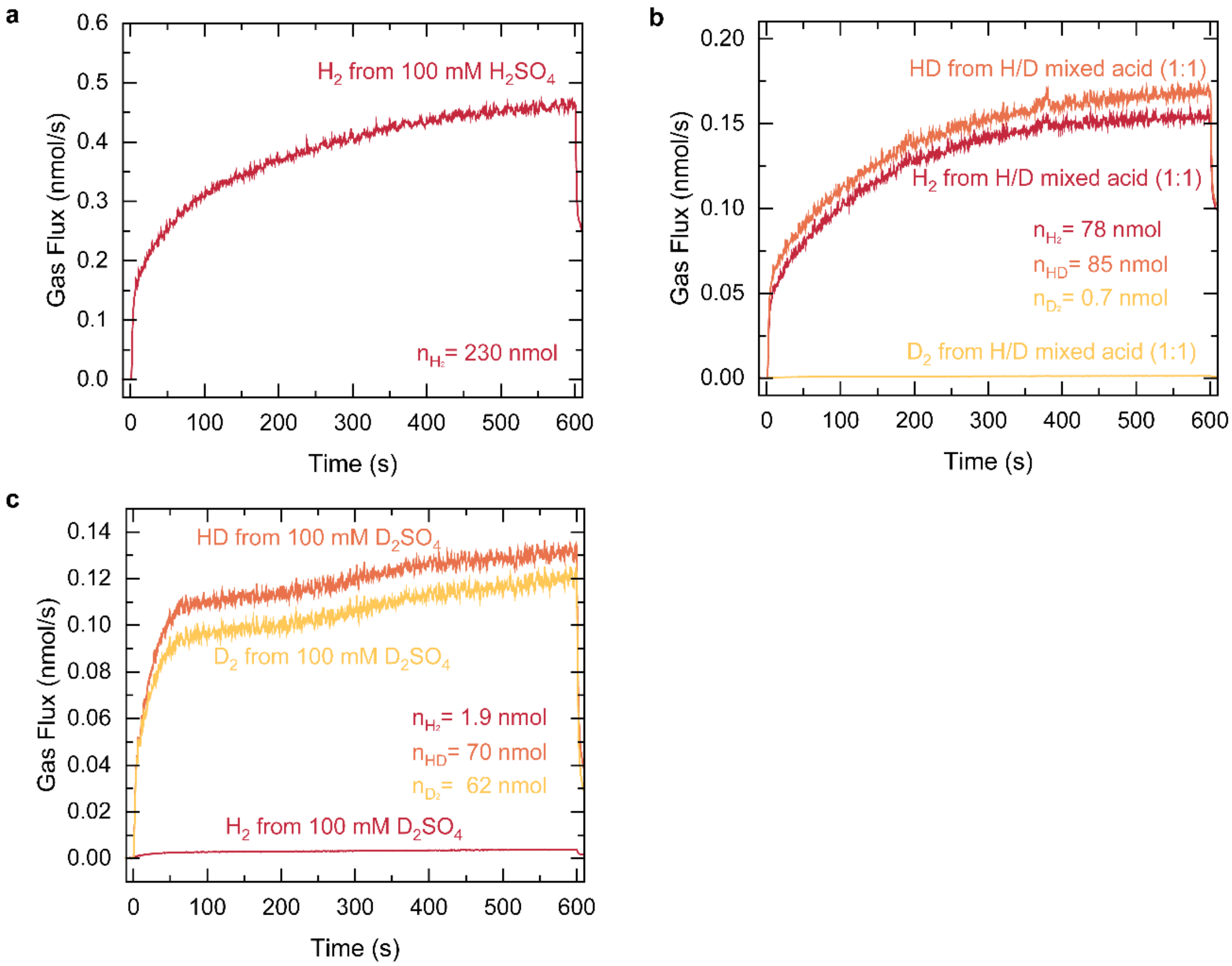

Fig. S6. Electrochemical mass spectrometry (ECMS) data collected during CP at -0.2 mA, showing **(a)** H_2 flux from 100 mM H_2SO_4 **(b)** H_2, HD and D_2 flux in 1:1 mixed H/D acid (50 mM H_2SO_4+ 50 mM D_2SO_4), and **(c)** 100 mM D_2SO_4. This flux is derived from Raw MS in ***Fig. S5***.

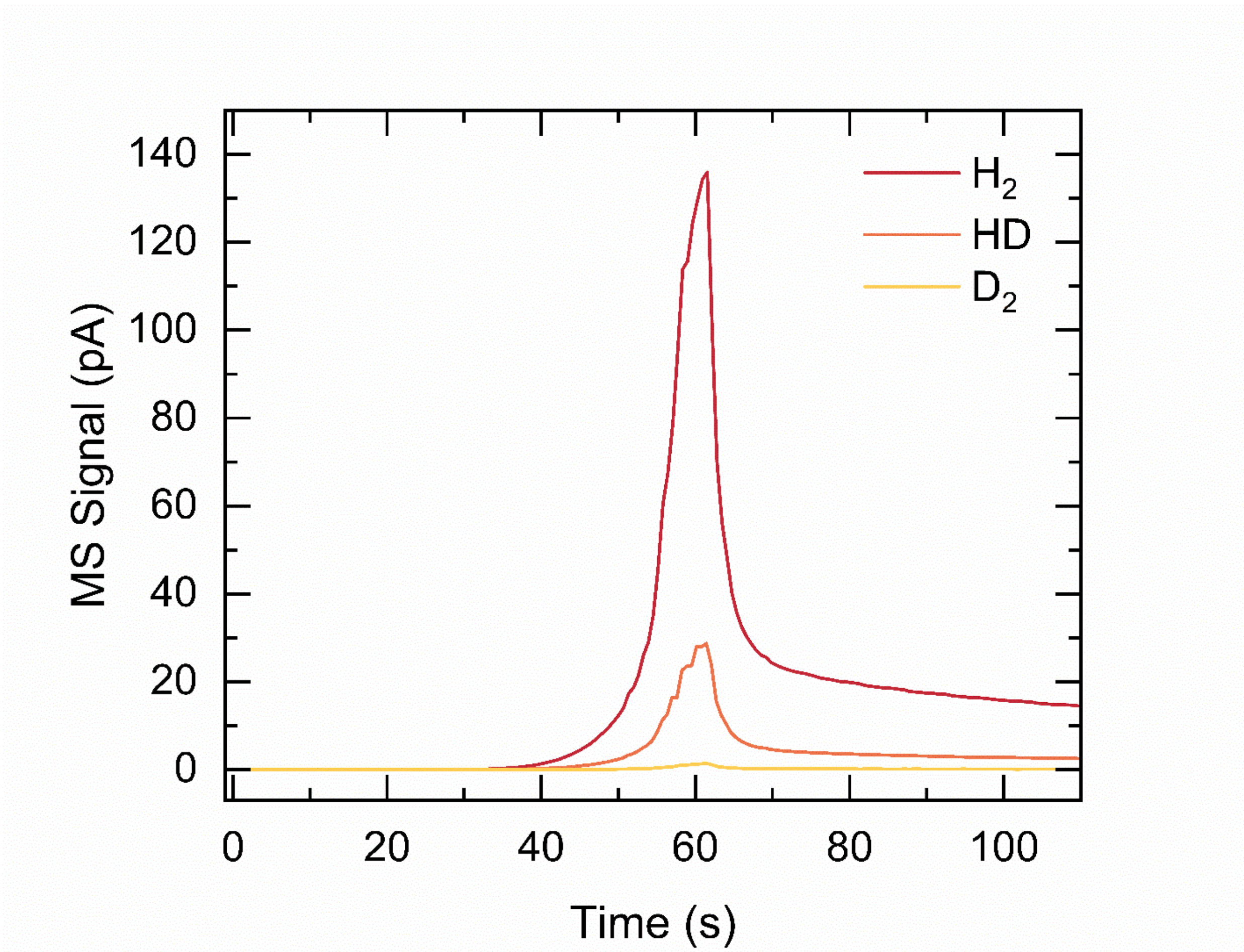

Fig. S7. Raw MS data collected during linear sweep voltammetry (LSV) at 5 mV s^{-1} for acid reduction in a 1:1 mixed H/D electrolyte (50 mM H_2SO_4 + 50 mM D_2SO_4).

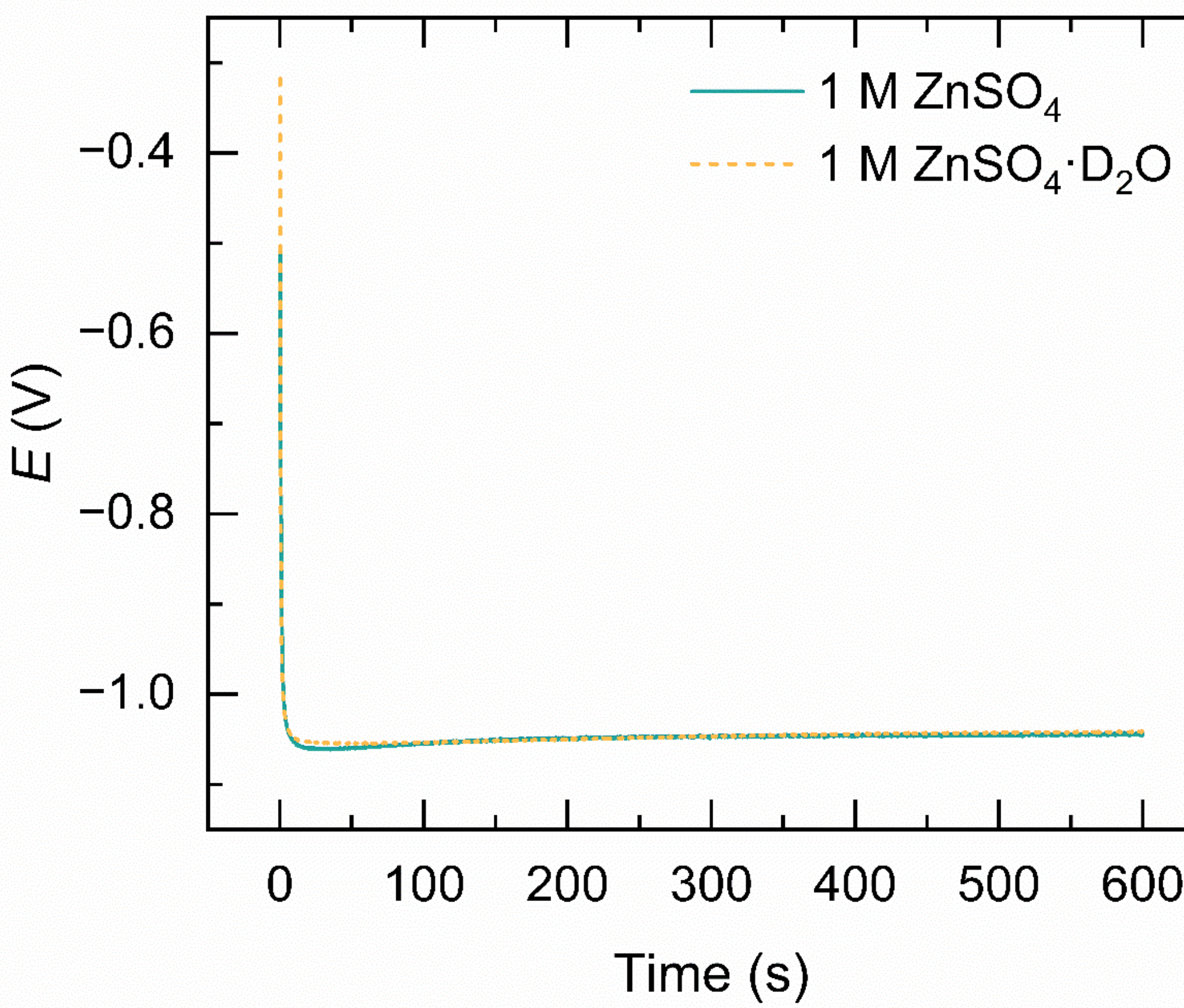

Fig. S8. Chronopotentiometry at -0.2 mA recorded using ECMS setup in a 3-electrode setup showing the real-time Zn electrodeposition.

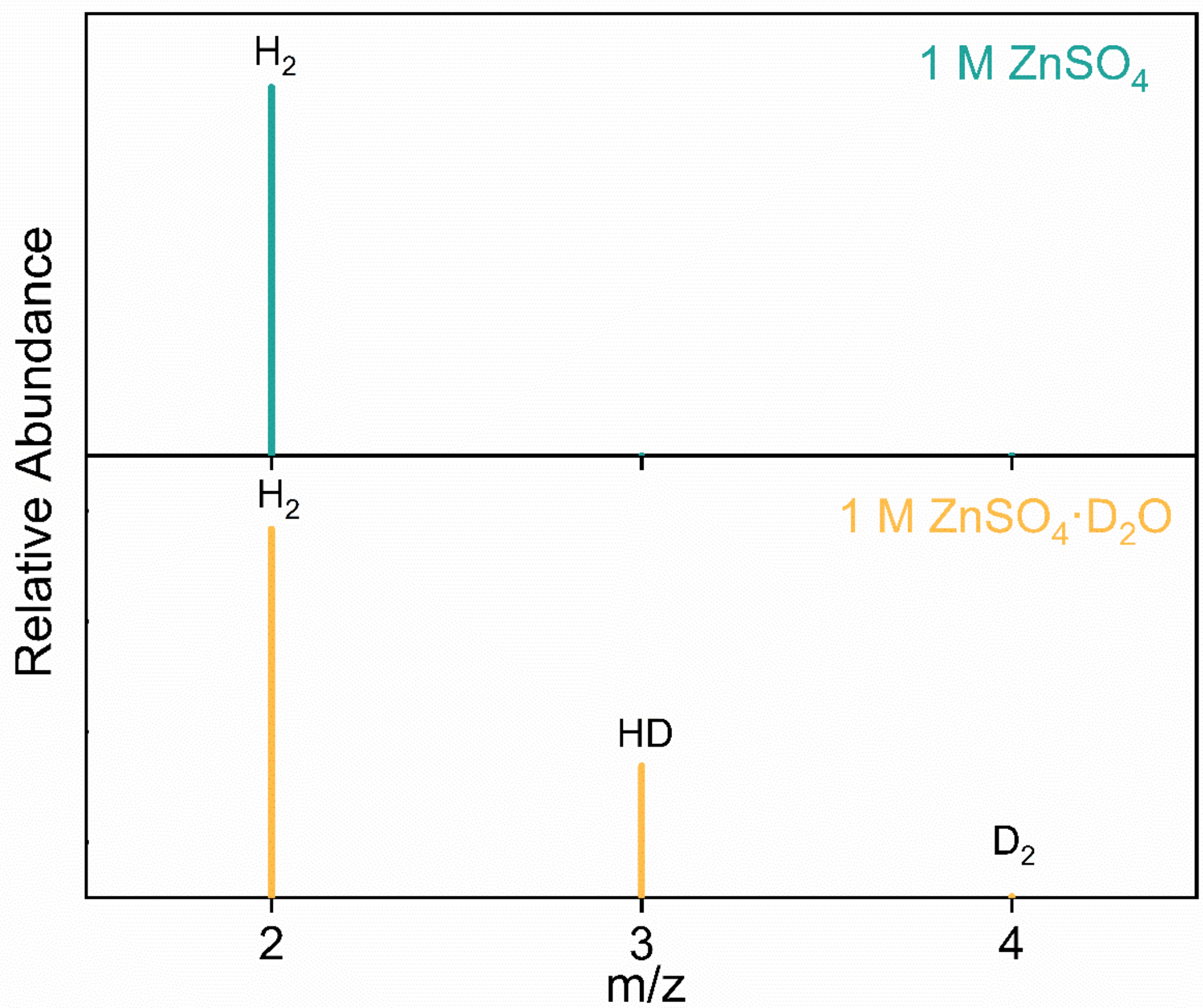

Fig. S9. Normalized mass spectrometry (MS) scan of evolved gases collected during chronoamperometry (CP) at -0.2 mA in H_2O-based (top, 1 M $ZnSO_4$) and D_2O-based (bottom, 1 M $ZnSO_4 \cdot D_2O$) electrolytes. Signals corresponding to m/z = 2 (H_2), 3 (HD), and 4 (D_2) are detected.

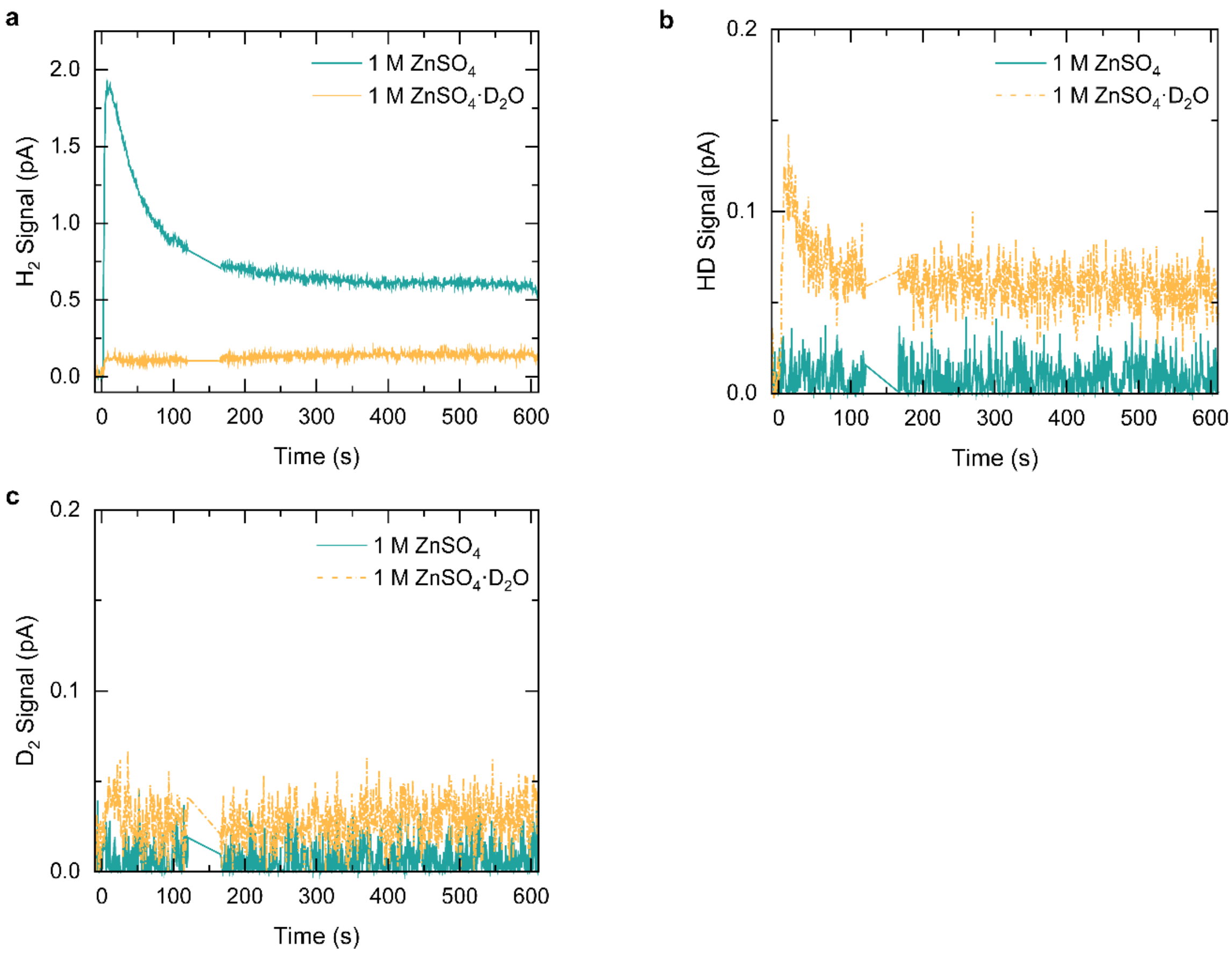

Fig. S10. Raw MS signals of **(a)** H_2, **(b)** HD, and **(c)** D_2 collected during CP at -0.2 mA in H_2O-based and D_2O-based 1 M $ZnSO_4$ electrolytes. The transient signal loss at early times arises from MS switching/transfer delays; the corresponding processed signals are presented in ***Fig. S9***.

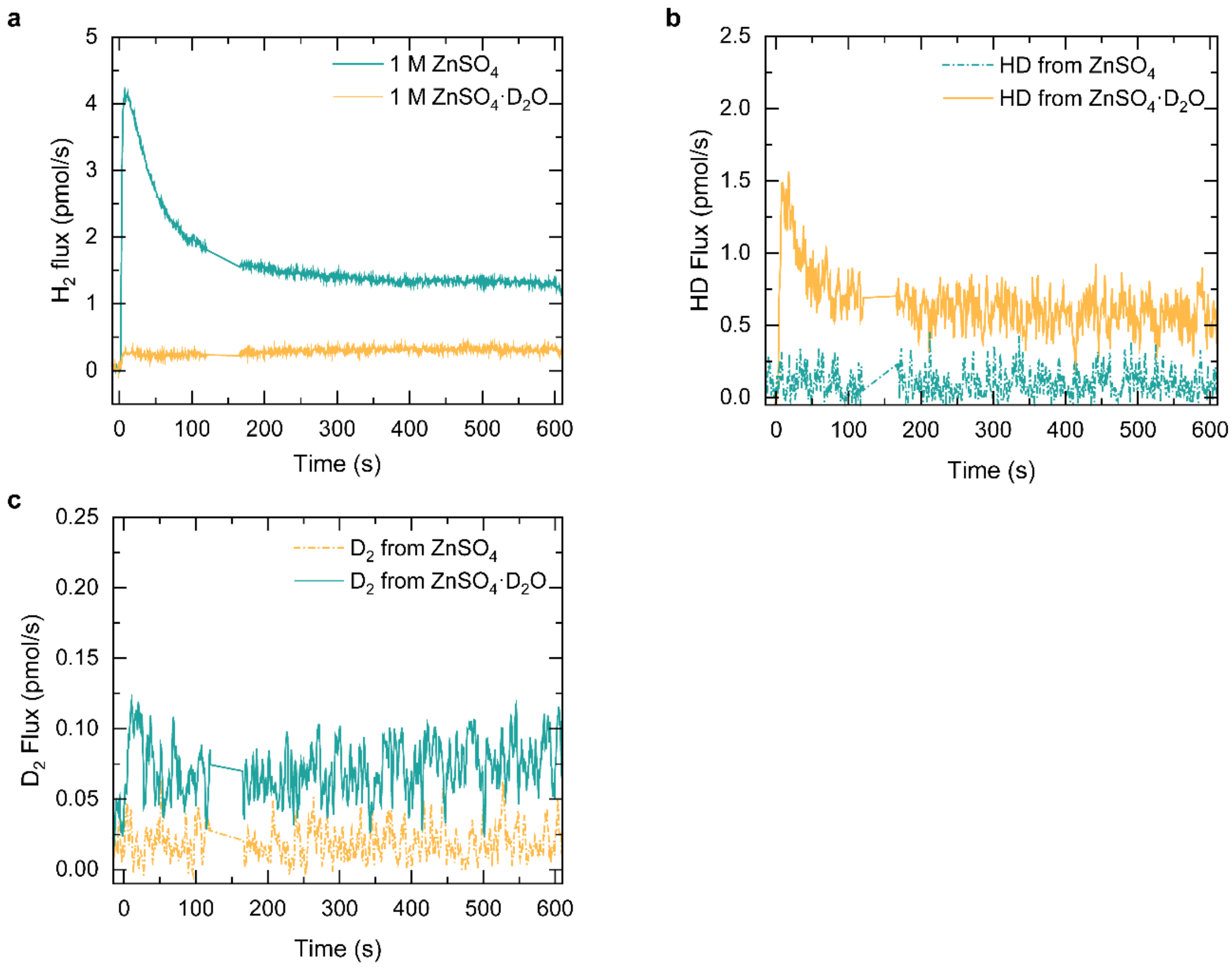

Fig. S11. Electrochemical mass spectrometry (ECMS) data collected during chronopotentiometry (CP) at -0.2 mA, showing **(a)** H_2, **(b)** HD flux and **(c)** D_2 flux in 1M $ZnSO_4$ and 1M $ZnSO_4$ in D_2O. This flux is derived from Raw MS, shown in ***Fig. S10***.

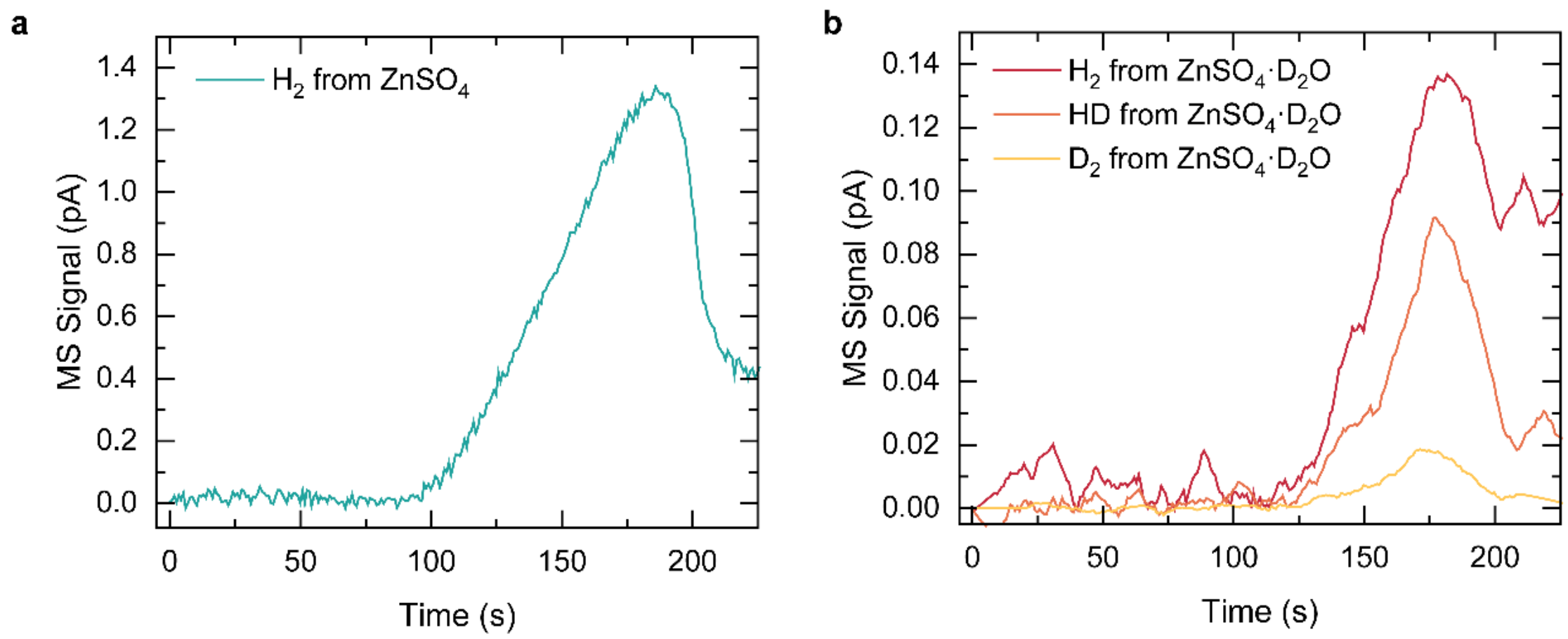

Fig. S12. Raw MS data collected during linear sweep voltammetry (LSV) at 5 mV s^{-1} for Zn reduction in **(a)** 1 M $ZnSO_4$ and **(2)** 1 M $ZnSO_4{\cdot}D_2O$.

Calibrations (all)

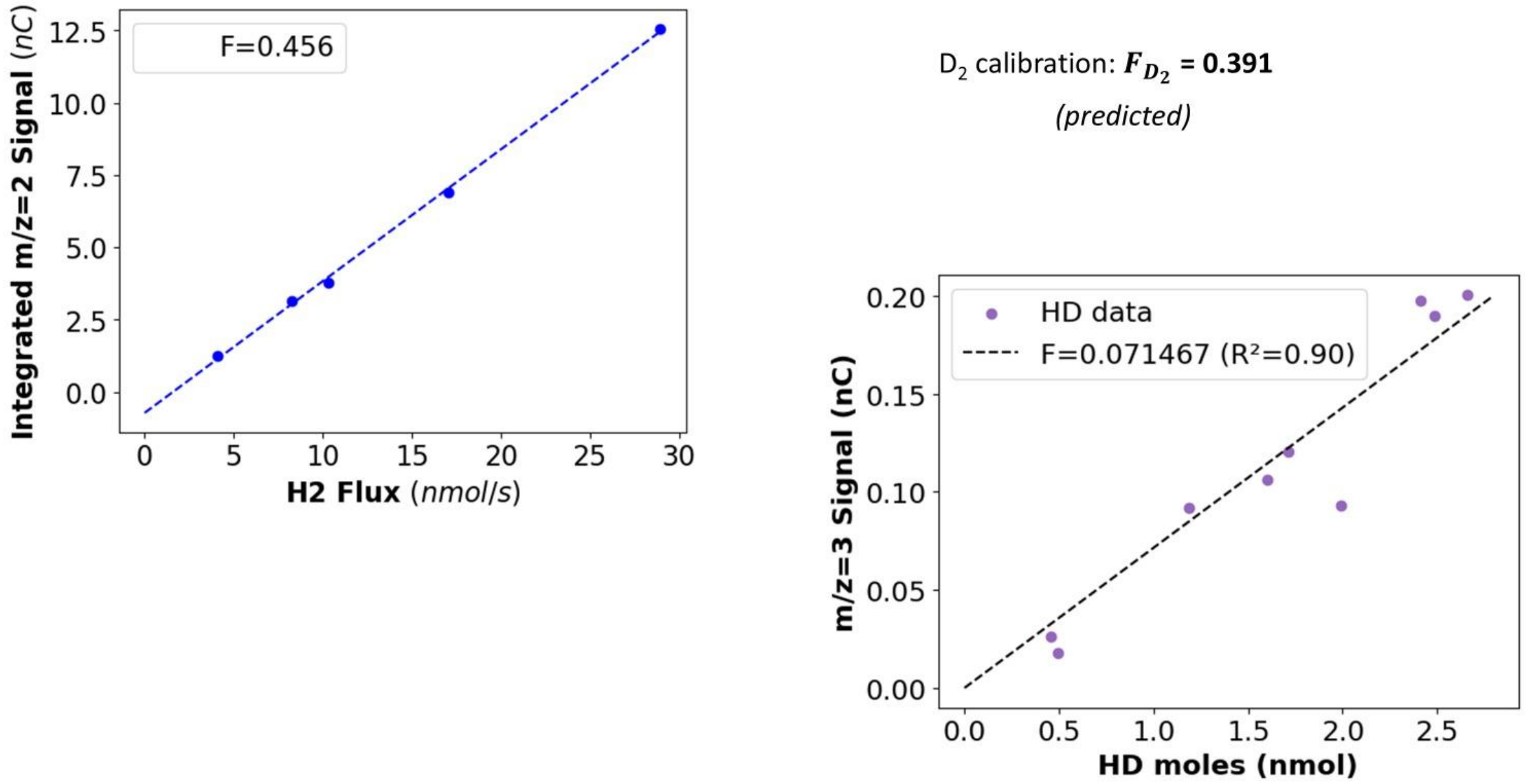

Fig. S13. Calibration curves for hydrogen isotopologues. Experimental calibration for H_2 (m/z = 2) obtained using a Pt electrode in 1 M $HClO_4$ under reductive chronopotentiometry. The integrated MS signal was plotted against the theoretical H_2 flux calculated from Faraday's law, yielding a calibration factor of F = 0.456 C/mol. The D_2 calibration factor (F = 0.391 C/mol) was predicted using ionization cross-section considerations based on literature values. Calibration for HD (m/z = 3) derived from a calculation shown above, with the integrated signal plotted against known HD amounts, resulting in a calibration factor of F = 0.0715 C/mol and $R^2 = 0.90$.

HD calibration determination

Assuming 2 electron processes for H2, D2 and HD formation
Assuming negligible H/D scrambling in ionization chamber

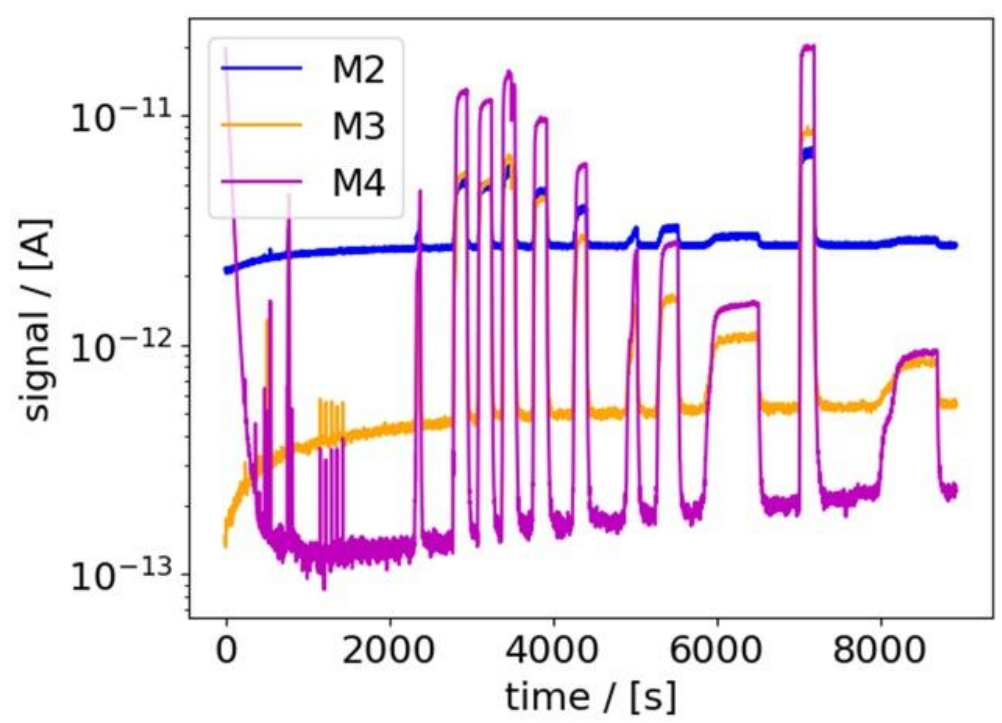

MS spectra obtained from varying CP in 1M D_2SO_4/D_2O electrolyte

For each CP step:
Electrons are going to all three faradaic reactions

$$i_{total} = i_{HER} + i_{DER} + i_{(H-D)ER}$$

$$flux_{H_2} = \frac{MS\ signal}{F_{H_2}} \qquad flux_{D_2} = \frac{MS\ signal}{F_{D_2}}$$

$$i_{HER} = flux_{H_2} \cdot nF \qquad i_{DER} = flux_{D} \cdot nF$$

$$i_{(H-D)ER} = i_{total} - (i_{HER} + i_{DER})$$

Calibration curve of m/z = 3 signal vs. $i_{(H-D)ER}$ will give F_{HD}

Fig. S14. Schematic and representative data illustrate the procedure for determining the calibration factor of HD (m/z = 3) in ECMS. Chronopotentiometry (CP) experiments were performed at varying current steps in 1 M D_2SO_4/D_2O electrolyte, while simultaneously recording the MS signals corresponding to H_2 (m/z = 2), HD (m/z = 3), and D_2 (m/z = 4). The total current at each CP step was assumed to be the sum of faradaic currents associated with HER (H_2), HDER (HD), and DER (D_2).

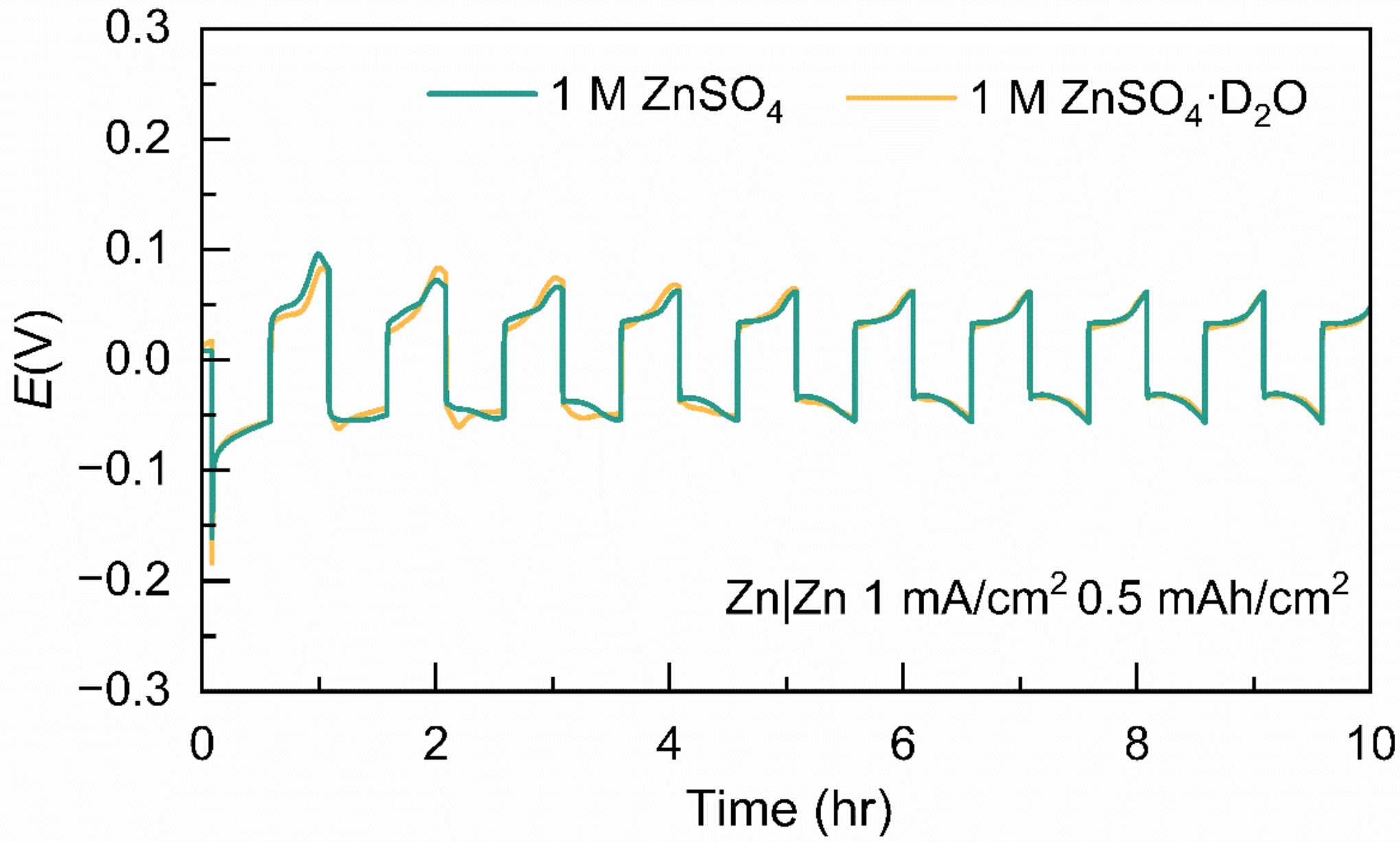

Fig. S15. First 10 cycles of Zn||Zn symmetric cell cycling at 1 mA cm^{-2} with a capacity of 0.5 mAh cm^{-2} in 1 M $ZnSO_4$ and 1 M $ZnSO_4 \cdot D_2O$ electrolytes.

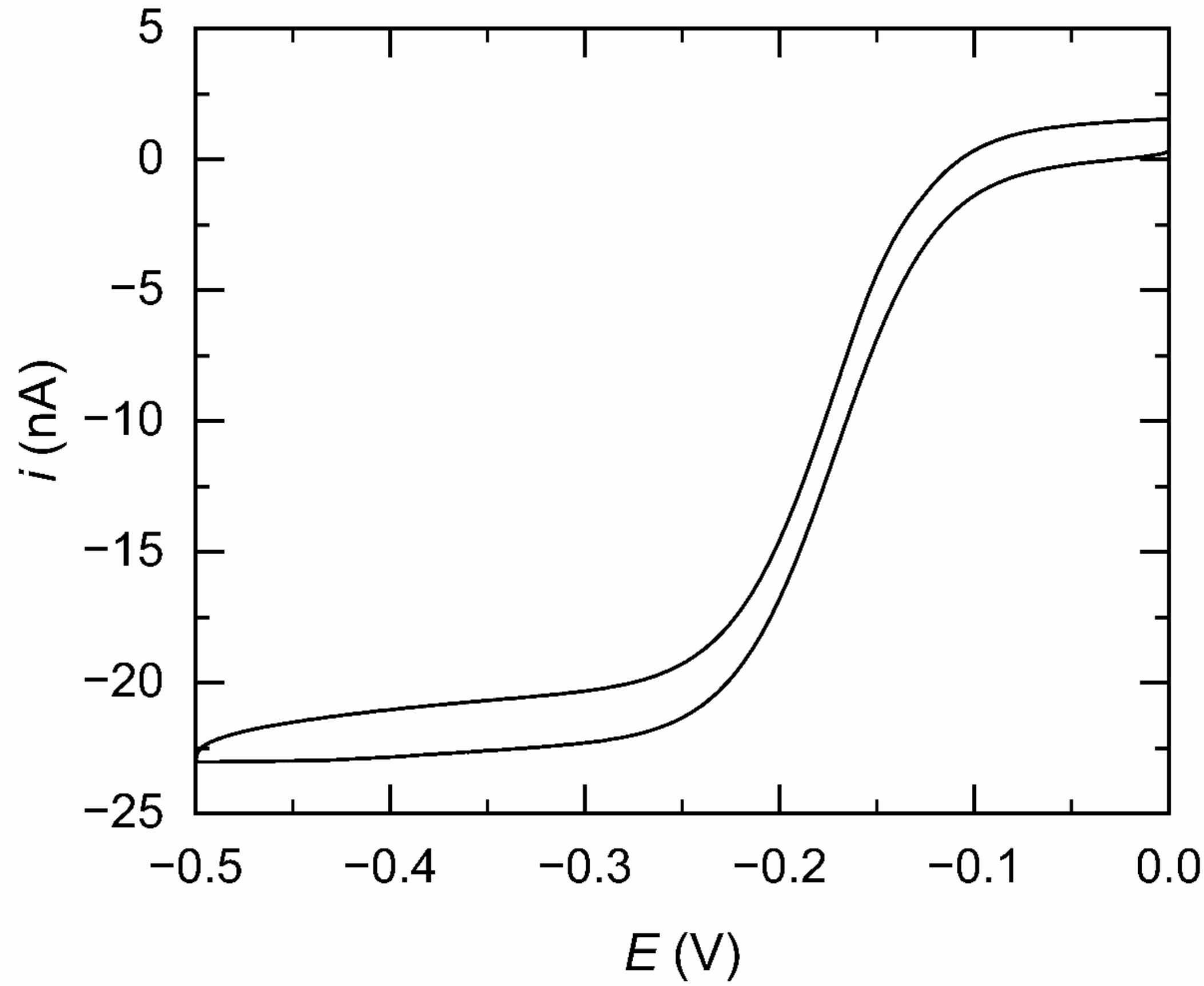

Fig. S16. Cyclic voltammogram obtained at a scan rate of 10 mV s^{-1} in 5 mM hexaammineruthenium(III) chloride, measured in a two-electrode setup employing a homemade W UME as the working electrode and Ag/AgCl in 1 M KCl as the counter/reference electrode.

**Scan rate, v (Vs^{-1})**	**j_0 in H_2O (mA/cm^2)**	**j_0 in D_2O (mA/cm^2)**
0.05	380	330
0.5	160	110
1	110	70
5	70	41
10	74	45
20	83	65
30	68	63
40	87	85
50	87	83
60	87	83

Table S1. Exchange current densities (j_0) of 1M $ZnSO_4$ in H_2O and D_2O, derived from Tafel analysis at all scan rates.

	**OCP**	**Crossover (50 mV s^{-1})**	**Crossover (60 V s^{-1})**
1M $ZnSO_4$	-1.003 V	-1.037 V	-1.056 V
1M $ZnSO_4 \cdot D_2O$	-1.004 V	-1.033 V	-1.057 V

Table S2. Open-circuit potentials (OCP) measured using a Zn working electrode, and crossover potentials extracted from slow-scan cyclic voltammetry (SSCV, 50 mV s^{-1}) and fast-scan cyclic voltammetry (FSCV, 60 V s^{-1}) on a tungsten ultramicroelectrode (UME) in 1 M $ZnSO_4$ electrolytes prepared with H_2O and D_2O (vs. Ag/AgCl).

**Scan rate, v (Vs^{-1})**	**j_0 H_2O (mA/cm^2)**	**j_0 50% D_2O (mA/cm^2)**	**j_0 D_2O (mA/cm^2)**
40	83	88	75
50	89	95	75
60	94	94	75

Table S3. Exchange current densities (j_0) extracted from fast-scan cyclic voltammetry (FSCV) at different scan rates for 1M $Zn(OTf)_2$ electrolytes prepared with H_2O, 50% D_2O, and D_2O.

References

1. Allen J. Bard, Larry R. Faulkner, Henry S. White. Electrochemical Methods: Fundamentals and Applications (2022).
2. D. T. Boyle, *et al.*, Transient Voltammetry with Ultramicroelectrodes Reveals the Electron Transfer Kinetics of Lithium Metal Anodes. *ACS Energy Lett.* **5**, 701–709 (2020).
3. M. A. Faisal, J. E. Dick, A Battery Researcher's Guide to Successful Ultramicroelectrode Fabrication for Fast-Scan Kinetic Analysis. *ACS Appl. Energy Mater.* **7**, 10326–10334 (2024).
4. A. Rana, *et al.*, How the Kinetic Balance Between Charge-Transfer and Mass-Transfer Influences Zinc Anode Stability: An Ultramicroelectrode Study. *Small Methods* **9** (2025).
5. K. Roy, A. Rana, J. N. Heil, B. M. Tackett, J. E. Dick, For Zinc Metal Batteries, How Many Electrons go to Hydrogen Evolution? An Electrochemical Mass Spectrometry Study. *Angew. Chem. Int. Ed.* **63**, e202319010 (2024).
6. K. Roy, *et al.*, How Solvation Energetics Dampen the Hydrogen Evolution Reaction to Maximize Zinc Anode Stability. *Adv. Energy Mater.* **14** (2024).
7. Søren Bertelsen Scott, "Isotope-Labeling Studies in Electrocatalysis for Renewable Energy Conversion – And the net CO_2 impact of this PhD Project," Technical University of Denmark (DTU). (2019).
